# A transcriptome-based classifier to identify developmental toxicants by stem cell testing: design, validation and optimization for histone deacetylase inhibitors

**DOI:** 10.1007/s00204-015-1573-y

**Published:** 2015-08-14

**Authors:** Eugen Rempel, Lisa Hoelting, Tanja Waldmann, Nina V. Balmer, Stefan Schildknecht, Marianna Grinberg, John Antony Das Gaspar, Vaibhav Shinde, Regina Stöber, Rosemarie Marchan, Christoph van Thriel, Julia Liebing, Johannes Meisig, Nils Blüthgen, Agapios Sachinidis, Jörg Rahnenführer, Jan G. Hengstler, Marcel Leist

**Affiliations:** Department of Statistics, TU Dortmund University, 44139 Dortmund, Germany; Centre for Organismal Studies, Heidelberg University, 69120 Heidelberg, Germany; Doerenkamp-Zbinden Chair for In Vitro Toxicology and Biomedicine, University of Konstanz, Box: M657, 78457 Konstanz, Germany; Konstanz Graduate School Chemical Biology KORS-CB, University of Konstanz, 78457 Konstanz, Germany; Institute of Neurophysiology, Center for Molecular Medicine Cologne (CMMC), University of Cologne, 50931 Cologne, Germany; Leibniz Research Centre for Working Environment and Human Factors, Technical University of Dortmund (IfADo), 44139 Dortmund, Germany; Institute of Pathology, Charité-Universitätsmedizin, 10117 Berlin, Germany; Integrative Research Institute for the Life Sciences, Institute for Theoretical Biology, Humboldt Universität, 10115 Berlin, Germany

**Keywords:** Hazard assessment, Neuronal development, Alternative testing, Cytotoxicity, Transcriptomics, Developing central nervous system

## Abstract

Test systems to identify developmental toxicants are urgently needed. A combination of human stem cell technology and transcriptome analysis was to provide a proof of concept that toxicants with a related mode of action can be identified and grouped for read-across. We chose a test system of developmental toxicity, related to the generation of neuroectoderm from pluripotent stem cells (UKN1), and exposed cells for 6 days to the histone deacetylase inhibitors (HDACi) valproic acid, trichostatin A, vorinostat, belinostat, panobinostat and entinostat. To provide insight into their toxic action, we identified HDACi consensus genes, assigned them to superordinate biological processes and mapped them to a human transcription factor network constructed from hundreds of transcriptome data sets. We also tested a heterogeneous group of ‘mercurials’ (methylmercury, thimerosal, mercury(II)chloride, mercury(II)bromide, 4-chloromercuribenzoic acid, phenylmercuric acid). Microarray data were compared at the highest non-cytotoxic concentration for all 12 toxicants. A support vector machine (SVM)-based classifier predicted all HDACi correctly. For validation, the classifier was applied to legacy data sets of HDACi, and for each exposure situation, the SVM predictions correlated with the developmental toxicity. Finally, optimization of the classifier based on 100 probe sets showed that eight genes (F2RL2, TFAP2B, EDNRA, FOXD3, SIX3, MT1E, ETS1 and LHX2) are sufficient to separate HDACi from mercurials. Our data demonstrate how human stem cells and transcriptome analysis can be combined for mechanistic grouping and prediction of toxicants. Extension of this concept to mechanisms beyond HDACi would allow prediction of human developmental toxicity hazard of unknown compounds with the UKN1 test system.

**Electronic supplementary material:**

The online version of this article (doi:10.1007/s00204-015-1573-y) contains supplementary material, which is available to authorized users.

## Introduction

Classification and grouping of toxicants is a major goal of toxicological risk assessment, next to the *de novo* prediction of hazard for entirely new compounds (Gocht et al. 2015). Such methods are particularly useful when testing for reproductive and developmental toxicity due to (1) a large backlog of substances to be evaluated, (2) an especially high demand in resources and animals and (3) the difficult issue of data interpretation in this field. Moreover, it is well established that the developing central nervous system is particularly susceptible to chemicals (Smirnova et al. 2014b; van Thriel et al. 2012). Currently, developmental neurotoxicity is tested using labour-intensive in vivo experiments according to OECD test guidelines TG 426, which requires exposure of animals during gestation and lactation, followed by analyses for histopathological, functional and behavioural abnormalities in the offspring. As this in vivo test is too expensive for the analysis of thousands of untested but marketed chemicals, alternative tests are urgently needed to prioritize test compounds for further analysis by more extensive studies (Bal-Price et al. 2015; Leist et al. 2014).

To reach this goal, human embryonic stem cell (hESC)-based test systems have recently been developed (Bal-Price et al. 2012; Colleoni et al. 2011; Efthymiou et al. 2014; Harrill et al. 2011; Jagtap et al. 2011; Krug et al. 2013; Leist et al. 2008a; Meganathan et al. 2012; Pallocca et al. 2013; van Thriel et al. 2012; Wheeler et al. 2015; Zimmer et al. 2012, 2014). These test systems recapitulate different critical phases of embryonic development during which the differentiating cells can be exposed to chemicals. A particularly intensively studied phase is neural induction, when the neural ectodermal progenitor cells are formed. This phase can be recapitulated, using the cell system UKN1, which has recently been optimized for transcriptomics approaches (Balmer et al. 2012, 2014; Krug et al. 2013). In this in vitro system, the known developmental neurotoxicants valproic acid (VPA) and methylmercury have been shown to induce specific and reproducible gene expression patterns that can easily be distinguished from negative control compounds. Moreover, the system revealed concentration progression principles with (1) tolerated, (2) teratogenic but non-cytotoxic and (3) finally cytotoxic ranges, at similar concentrations as in humans (Waldmann et al. 2014).

A next challenge in the UKN1 test system development is the establishment of gene expression-based classifiers for compounds acting by similar mechanisms. Histone deacetylase inhibitors (HDACi) have been chosen as a class of model compounds in the present study, as they are known to cause neural tube defects in animals and humans (Balmer et al. 2012; Kadereit et al. 2012; Nau et al. 1991). Inhibition of histone deacetylases triggers large changes in the cellular transcriptome at in vivo relevant concentrations (Jergil et al. 2009; Krug et al. 2013; Smirnova et al. 2014a; Theunissen et al. 2012; Waldmann et al. 2014; Werler et al. 2011). Since VPA acts as a reversible inhibitor of enzyme activity, changes in the transcriptome can therefore be reversible. Indeed, it has been shown that up- or down-regulated genes in developing neuronal precursor cells can return to control levels after short-term exposure of 6 h. However, longer exposure period of 4 days, which covered critical time windows of development, led to transcriptional changes that were irreversible after washout of the toxicant (Balmer et al. 2014). Besides VPA, five further HDACi were studied, namely belinostat (PXD101), entinostat (MS-275), panobinostat (LBH589), vorinostat (SAHA) and trichostatin A (TSA). Although these compounds differ in their isoenzyme specificity (Khan et al. 2008), they all produce potent inhibition of major members of the HDAC family (HDAC-1, 2, 4, 6) and have all been developed for a similar indication (tumour chemotherapy). Therefore, the six HDACi can be considered as a relatively homogeneous group with respect to their mode of action. ‘Mercurials’ were selected for the second group, which were defined by the commonality of having one mercury atom in their chemical structure: methylmercury chloride (MeHg), thimerosal, mercury(II)chloride (HgCl_2_), mercury(II)bromide (HgBr_2_), 4-chloromercuribenzoic acid (PCMB) and phenylmercuric acetate (PMA). These compounds form a more heterogeneous group than the aforementioned HDACi, ranging from inorganic salts, such as HgCl_2_ with ionized Hg^2+^ and small charged organomercurial ions (methyl-Hg^+^; ethyl-Hg^+^ from thimerosal), to completely organic structures (PCMB). All mercury compounds react with thiol groups and can therefore modify proteins with free cysteine groups (Bahr and Moberger 1954; Halsey 1955; Pekkanen and Sandholm 1971) leading to oxidative stress, inhibition of protein synthesis and disruption of calcium homeostasis (Suppl. Fig. S1). The most studied mercurial compound is MeHg because of the catastrophic endemic diseases caused by ingestion of MeHg-contaminated food (Choi 1989; Ekino et al. 2007; Harada 1995). It is also known to cause neural tube defects and other developmental disturbances, and as a result is considered a ‘gold standard’ compound of human developmental toxicity (Grandjean and Herz 2011; Robinson et al. 2011).

The aim of the present work was to study (1) whether the six HDACi can be recognized as a homogeneous group based on gene array data, (2) whether the alterations they induce can be differentiated from those caused by mercurials and (3) whether a classifier can be constructed based on a support vector machine. Finally, the gene set required for correct classification was optimized and reduced to a minimum to facilitate routine testing. We report that an eight-gene-based classifier correctly identifies all tested HDACi. With this classifier, it should be possible to predict with a high probability whether an unknown compound can be classified as an HDACi.

## Materials and methods

### Materials

Gelatin, putrescine, selenium, progesterone, apotransferrin, glucose and insulin were obtained from Sigma (Steinheim, Germany). Accutase was from PAA (Pasching, Austria). FGF-2 (basic fibroblast growth factor), noggin and sonic hedgehog were obtained from R&D Systems (Minneapolis, MN, USA). Y-27632, SB-43154 and dorsomorphin dihydrochloride were from Tocris Bioscience (Bristol, UK). MatrigelTM was from BD Biosciences (Massachusetts, USA). All cell culture reagents were from Gibco/Invitrogen (Darmstadt, Germany) unless otherwise specified.

### Neuroepithelial differentiation

**Fig. 1:**
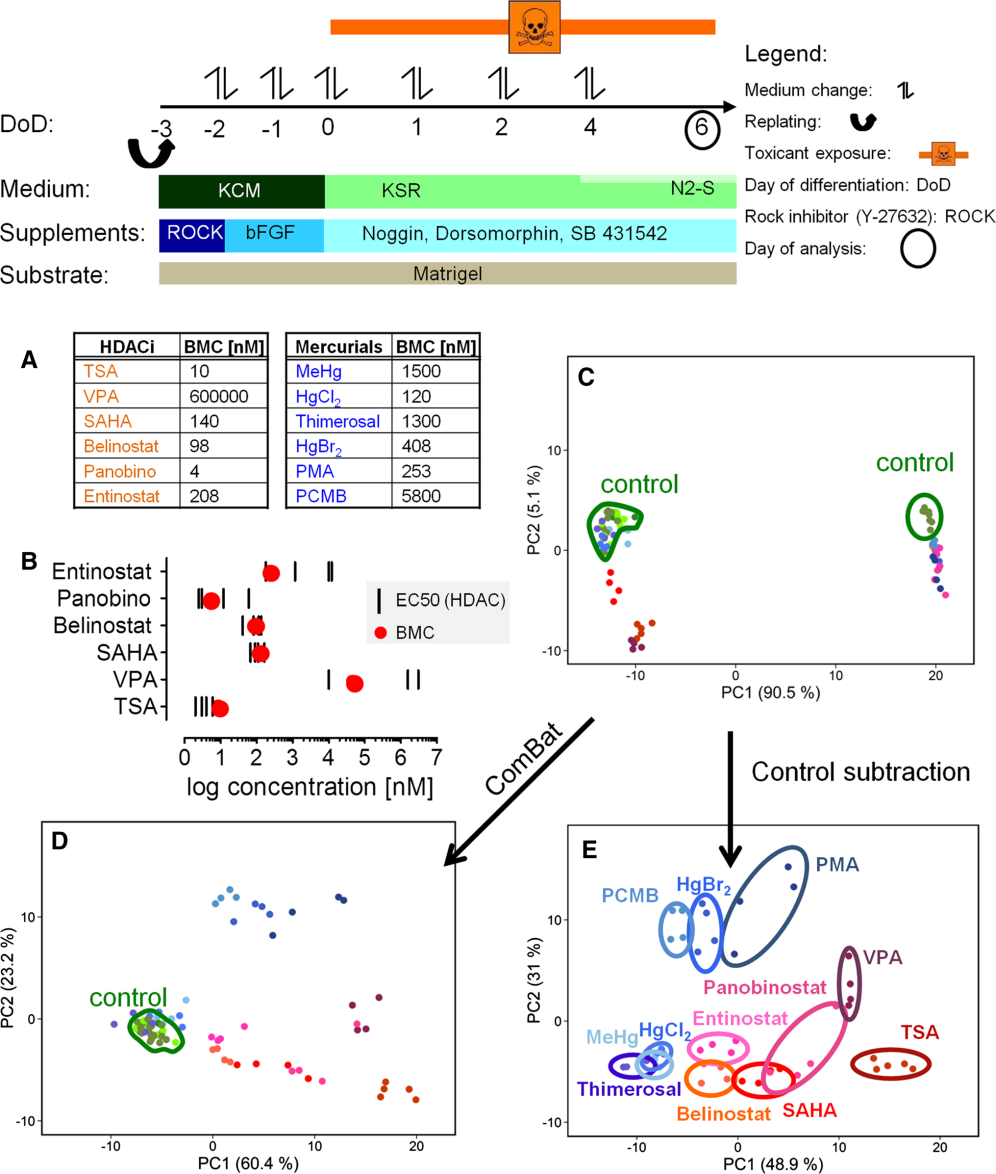
Data structure of transcriptome changes triggered by histone deacetylase inhibitors (HDACi) and mercurials in human stem cells differentiating to neuroectoderm. Stem cells were differentiated towards neuroectodermal progenitor cells within 6 days of differentiation (DoD6) as indicated on *top*. **a** The highest non-cytotoxic concentration [corresponding to EC10(cytotoxicity)] of all test compounds was determined in a viability assay. This ‘benchmark concentration’ (BMC) was used for obtaining transcriptome data of HDACi and mercurials in this study. The BMC was calculated, based on concentration–response curves of three independent experiments. **b** EC50 data for inhibition of HDAC isoforms 1, 2, 4, 6 were retrieved from the literature (Khan et al. 2008). They are indicated by a *black line*, and the respective BMC in our study is indicated as *red dot*. **c** The data structure of all transcriptome data sets was dimensionality-reduced and presented in form of a 2D principle component analysis (PCA) diagram. Data show a typical batch effect (offset of controls) which segregated with measurements from different sets of biological samples. **d** The ComBat batch correction algorithm perfectly aligned the controls and led to compound-wise clustering on the PCA diagram. **e** Alternatively, respective control values were subtracted from treated samples. This simple manipulation also led to a satisfying batch correction and clustering of data points according to toxicants. Each point represents one experiment (=data from one microarray), and the colour coding (labelled in **e** for data in **c**–**e**) indicates the compound used in the experiment; panobino, panobinostat (colour figure online)

The human pluripotent stem cell line H9 (Thomson et al. 1998) was cultured according to standard protocols and differentiated into neuroepithelia progenitors (NEP) as described earlier (Balmer et al. 2012; Krug et al. 2013; Shinde et al. 2015) and as shown in Fig. 1. The H9 hESC line (WA09 line) was obtained from WiCell (Madison, WI, USA). Importation of cells and subsequent experiments was authorized under license # 170-79-1-4-27 (Robert Koch Institute, Berlin, Germany). Differentiation of the H9 cells towards NEP was based on dual SMAD inhibition (Chambers et al. 2009) using a combination of 35 µM noggin and 600 nM dorsomorphine together with 10 µM SB-431642. This was used to prevent BMP and TGF signalling and thus achieve a highly selective neuroectodermal lineage commitment. For handling details, see supplemental methods of (Balmer et al. 2012).

### Experimental exposure and resazurin viability assay

During differentiation, cells were treated with the respective HDACi and mercurials as indicated in Fig. 1. On DoD0, medium was prepared as described in Balmer et al. (2012) and supplemented with the indicated concentrations of the respective HDACi or mercurials. All concentrations were prepared from a stock solution. Medium, supplemented with the toxicant, was changed on DoD1, 2 and 4. In order to determine cytotoxicity, a resazurin cell viability assay was performed on DoD6 exactly as described previously (Krug et al. 2013; Stiegler et al. 2011). A detailed list of the 6 HDACi and 6 mercurials and their solvent and stock concentration is shown in the table below.

**Table:** 

Compound	Solvent	Stock concentration (mM)	Catalog #	Provider
Trichostatin A (TSA)	DMSO	5	T1952	Sigma
Valproic acid (VPA)	Water	600	P4543	Sigma
Vorinostat (SAHA)	DMSO	50	SML 0061	Sigma
Belinostat (PXD101)	DMSO	100	S1085	Selleckchem
Panobinostat (LBH589)	DMSO	100	S1030	Selleckchem
Entinostat (MS-275)	DMSO	50	Cay-13284-25	Biomol
Methylmercury (MeHg)	10 % ethanol	10	442534	Sigma
Thimerosal	Water	100	T4687	Sigma
Mercury(II)chloride (HgCl_2_)	Water	200	203777	Sigma
Mercury(II)bromide (HgBr_2_)	Water	10	437859	Sigma
Phenylmercuric acetate (PMA)	Water	10	P27127-25G	Sigma
4-Chloromercuribenzoic acid (PCMB)	Water	100	C5913-5G	Sigma

For determination of the benchmark concentration (BMC), each toxicant was tested at multiple concentrations (exemplified in suppl. Fig. S2) above and below the BMC. Public domain software (PROAST) was used for curve fitting and BMC calculation. For the microarray analysis, cells were treated with only a single concentration until DoD6. Cell viability was then determined using the resazurin cell viability assay, the media removed, and cells stored in 500 µl RNAProtect Reagent (Qiagen) until RNA isolation.

### Affymetrix DNA microarray analysis

RNA was extracted, and Affymetrix chip-based DNA microarray analysis (Human Genome U133 plus 2.0 arrays) was performed as described earlier (Krug et al. 2013).

The corresponding raw CEL files of the Affymetrix chips are publicly available under GEO accession number GSE71127. The differentially expressed probe sets for each compound, including fold changes and *p* values of the limma *t* test are given in supplementary tables provided in an Excel file format.

### Support vector machine-based classification

A support vector machine (Cortes 1995) algorithm with linear kernel was used for the discrimination between histone deacetylase inhibitors (HDACi) and mercurials gene array data sets (R package ‘e1071’, functions ‘tune.svm’, ‘svm’ and ‘predict.svm’). After subtracting the corresponding controls, the number of variables was reduced as follows. For every classification analysis, the 100 probe sets (PS) with highest variance within the training set were selected from the original 54,000 PS. Then the function ‘tune.svm’ was applied for the optimization of hyperparameters (with *C* = 2^*i*^ with *i* = −5, −4, … 1, 2), the function ‘svm’ with the option ‘probability = TRUE’ was used for building the classification model, and the function ‘predict.svm’ was used to obtain, for each replicate of the compounds in the test set, the probabilities that they belong to mercurials or HDACi. First, the SVM optimized the decision boundary between the classes. Then a logistic regression with direction orthogonal to the decision boundary was applied to calculate the probabilities.

In order to avoid over-fitting and to analyse the generalization properties of fitted models, we split the original set of 12 compounds in training and test sets. For each split, we used the training set to build the classifier that includes (1) reduction of the variables, (2) optimization of a hyperparameter C and (3) determination of a classification rule. Then we assessed the accuracy on the test set with a ‘leave-out concept’ as follows. We performed a stability analysis by choosing different sets of compounds as test set. The number of compounds in the test set assumed values from 1 to 4, where a value of 1 corresponds to leave-one-out cross-validation. The analysis with larger numbers (e.g. 3 for leave-three-out) helps identify compounds that are essential for correct classification.

### Construction of a transcription factor network

We downloaded raw data for the microarray samples referenced in the manually curated CellNet tissue atlas (Cahan et al. 2014) and combined them with data from test systems UKN1 and UKK (Balmer et al. 2014; Krug et al. 2013; Waldmann et al. 2014). To obtain the expression matrix, the samples were normalized together using RMA implemented in the R package oligo. The co-expression network was constructed in two steps using functions from the parmigene package for R. First, the mutual information matrix was computed by applying the function knnmi, all with parameter *k* = 9 on the expression matrix. Then, we applied the CLR function from the parmigene package which implements the CLR algorithm. The co-expression network was subsequently restricted to genes annotated as transcription factors (TFs) in the Animal Transcription Factor Database (ATFDB, [http://www.bioguo.org/AnimalTFDB/index.php]). The overlap of the genes detected by the Affymetrix array and the ATFDB was 1300 genes. Links were drawn only for pairs of TFs with a score in the top 0.1 % of all scores of co-expression. This yielded 1690 predicted interactions between 847 TFs. Nodes were arranged in the network according to the Fruchterman and Reingold’s force directed placement algorithm provided by the R package sna with area parameter area = 10^9^.

## Representation of UKN1 genes and HDAC consensus genes on the TF network

Communities of network nodes were determined by the fastgreedy community function of the R package igraph. Only the top 18 largest communities (=clusters) were analysed for enrichment of GO biological process annotations. The enrichment analysis was performed with the R package topGO using the classic method and fisher test statistic. We selected representative terms to assign names to each community by applying the following procedure: (1) the over-represented GO terms were identified for each community and sorted in ascending order according to their *p* value; (2) the top GO terms were examined, and 1–2 key words that appeared representative for the overrepresented GO terms were selected; (3) these key words were used for naming of the communities (TF clusters); we restricted the selection to GO terms with an unadjusted over-representation *p* value of <0.05, apart from two cases, where no GO terms were overrepresented. These contained a large number of little-characterized zinc finger TF and were summarized under the name ‘unspecified cellular function and signalling’. The clusters annotated in this way were overlayed with HDAC consensus genes or TF genes known to be up-regulated on DoD6 versus DoD0. Alternatively, all HDACi consensus genes were pooled (up + down), and overrepresented TF were determined based on the network-based predicted interactions. Overrepresented TF were overlayed with the generic TF network.

### Caspase-3 inhibition assay by mercurials

Recombinant human caspase-3 (Millipore; CC-119) (0.25 U/200 µl reaction volume) in 50 mM Hepes, pH 7.4 containing 1 % sucrose and 0.1 % Chaps was treated with the respective mercurials at 37 °C for 20 min. Caspase-3 activity was then determined by the addition of the substrate *N*-acetyl-Asp-Glu-Val-Asp-7-amido-4-trifluoromethyl coumarin (NAc-DEVD-afc) (50 µM). Formation of free afc was assessed by fluorescence detection (*λ*_ex_: 385 nm; *λ*_em_: 505 nm) at 1 min intervals over 20 min (Gerhardt et al. 2001; Latta et al. 2000; Volbracht et al. 1999).

### Statistical analyses

The following analyses were performed using the statistical programming language ‘R-version 3.1.1’. For the normalization of the entire set of 85 Affymetrix gene expression arrays, the Extrapolation Strategy (RMA+) algorithm (Harbron et al. 2007) was used that applies background correction, log_2_ transformation, quantile normalization and a linear model fit to the normalized data in order to obtain a value for each PS on each array. As reference, the normalization parameters obtained in earlier analyses (Krug et al. 2013) were used. After normalization, the difference between gene expression and corresponding controls was calculated (paired design). Differential expression was calculated using the R package limma (Smyth et al. 2005). Here, the combined information of the complete set of genes is used by an empirical Bayes adjustment of the variance estimates of single genes. This form of a moderated *t* test is abbreviated here as ‘limma *t* test’. The resulting *p* values were multiplicity-adjusted to control the false discovery rate (FDR) by the Benjamini–Hochberg procedure (Benjamini 1995). As a result, for each compound a gene list was obtained, with corresponding estimates for log fold change and *p* values of the limma t test (unadjusted and FDR-adjusted).

#### Correction of batch effects

Non-biological experimental variation is known as batch effect and commonly observed across batches of microarray experiments. For batch correction, various approaches have been suggested in the literature (Scherer 2009). In this analysis, we used ComBat (Johnson et al. 2007) that was shown to be superior over other approaches (Chen et al. 2011). ComBat estimates parameters for location and scale adjustment for each batch and for each PS. As an alternative approach to reduce a possible batch effect and to remove the possible influence of, e.g., different solvents, the log2-transformed expression values of untreated samples were subtracted from the log2-transformed values of the corresponding matched treated samples from the same experiment (paired design). Thus, all treated samples were normalized to express fold changes relative to their controls. In cases when two controls were available for one treatment sample, the means of the controls were formed and then subtracted. For the adjustment of the values of a specific gene, the algorithm uses information of all genes on the array according to an Empirical Bayes framework (R package ‘sva’, function ‘ComBat’).

### Identification of consensus genes

A gene was defined as significantly deregulated by a specific compound if at least one annotated probe set was significantly deregulated (fold change >1.5 and adjusted *p* value of limma *t* test <0.05). A gene was defined as ‘consensus’ gene if it was significantly up- or down-regulated by at least 4 compounds of HDAC inhibitors or mercurials.

### Gene set enrichment analysis

The Gene Ontology group enrichment analysis was performed using ‘R-version 3.1.1’ with the ‘topGO’ package (Alexa 2010), and only results from the ‘biological process’ ontology were kept. Transcription factor binding site (TFBS) enrichment analysis was performed using the oPOSSUM web tool (Ho Sui et al. 2007) and based on JASPAR database (Portales-Casamar et al. 2010).

### KEGG pathway analysis

The analysis was conducted online using DAVID (Database for Annotation, Visualization and Integrated Discovery) (da Huang et al. 2009); the indicated *p* value represents the EASE Score, a modified Fisher’s exact *p* value, for gene-enrichment analysis.

## Results

### Data structure of transcriptome changes triggered by histone deacetylase inhibitors (HDACi) and mercurials

All experiments were performed under standard UKN1 test conditions, as described earlier (Krug et al. 2013). The twelve test compounds were tested under toxicologically comparable conditions, i.e. at their respective highest non-cytotoxic concentrations. This benchmark concentration (BMC) was determined experimentally using the UKN1 test system. The concentrations used for microarray analysis reduced viability of the stem cells after 6-day incubation by ≤10 % (Fig. 1a, suppl. Fig. S2). This anchoring of the BMC, which was then used for gene expression analysis, to the respective cytotoxicity of each toxicant correlated well with the biochemical mode of action within the group of HDACi. The BMC overlapped with the known EC50 values for HDAC inhibition. A similar mechanistic correlation was not possible for the group of mercurials due to their heterogeneous and less-defined mode of action (Suppl. Fig. S1).

Four independent experiments [except for TSA (*n* = 5) and MeHg (*n* = 5), and controls (*n* = 35)] were performed for each compound, and one Affymetrix chip was analysed per experiment, resulting in four genome-wide expression profiles per compound. To obtain an overview of the data structure (six HDACi, six mercurial and corresponding controls) of the 85 microarray data sets, the raw data were normalized and used for a principal component analysis (PCA). Visualization of the data along the first two principal components (PC) indicated two distinct clusters. These correlated with the data of measurement of the microarrays and thus represented a typical batch effect in this type of experiment. The two batches were differentiated along PC1, while the toxicant effect was mainly along PC2 (Fig. 1c). This was a favourable situation for the use of batch correction algorithms, and accordingly, the batch effect was perfectly removed by the ComBat algorithm. Visualization of the corrected data set showed that all HDACi were shifted to the lower right-hand side of the PCA plot (relative to controls). The position of the mercurials was more heterogeneous, with three clustering close to the controls, while the others shifted to the upper right (Fig. 1d). As alternative data correction, we used an approach that was previously successfully used in a similar experiment with microarrays from developing stem cells: the corresponding untreated controls were subtracted from each treatment data set (Krug et al. 2013). After this procedure, the PCA diagram was similar to the one achieved after ComBat correction (Fig. 1e). Therefore, only control-subtracted data sets, without further batch correction were used for all further analysis of toxicants.

### Overview of transcriptional changes induced by HDACi and mercurials

**Fig. 2:**
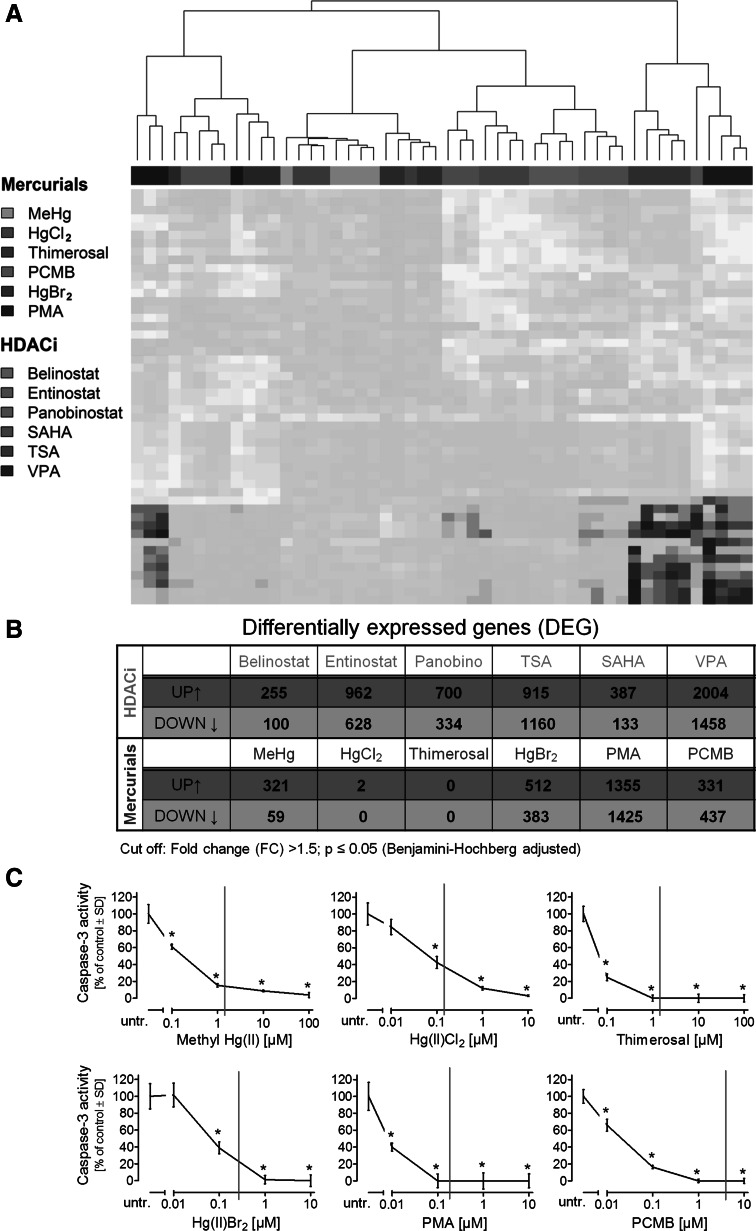
Characterization of transcriptional changes induced by HDACi and mercurials. **a** Differentiating cells were treated for 6 days by toxicants (four samples per compound; as in Fig. 1) before RNA was prepared and gene expression was measured on Affymetrix microarrays. The 50 genes with highest variance between all samples were selected for clustering (=clustering set). Then, all samples were clustered (Euclidean distance) on the basis of gene expression values for this set. The results are represented as heatmap with each row representing one gene, and the colour of each square indicating the absolute gene expression level (*blue* low; *green* middle; *yellow* high). **b** Number of differentially expressed genes (DEG) after exposure to toxicants compared to untreated controls (detailed data are shown in supplemental material). **c** Recombinant active caspase-3 was incubated for 30 min with respective mercurials at indicated concentrations. Then, the enzymatic activity was determined by a fluorometric assay. The caspase activity is represented in percentage relative to untreated control enzyme. The BMC of the respective mercurial (used in this study for microarray analysis) is indicated by a *red line*; data are mean ± SEM; *n* = 3; panobino, panobinostat (colour figure online)

For a first comparison, the probe sets (PS) with the highest variability across all toxicants (after subtraction of controls) were determined (50 PS) and used for cluster analysis. Two groups each of mercurials (weak/strong gene regulators) and HDACi (TSA and VPA/the other four) emerged. The data indicated that HDACi can be separated from mercurials and that more powerful statistical approaches should be able to unbiasedly separate the toxicant groups (Fig. 2a). As a basis for such follow-up work, all differentially expressed genes (DEG) were identified (Fig. 2b). Amongst the HDACi, belinostat and SAHA clearly deregulated less genes (about 350–500) than the other four members of the group (1000–3500 genes). Amongst the mercurials, HgCl_2_ and thimerosal had little effect on the transcriptome, and MeHg, HgBr_2_ and PCMB deregulated about 400–900 genes, and PMA about 2800 genes. Thus, the quantitative extent of transcriptome deregulation was very heterogeneous within the toxicant groups, showed some overlap between HDACi and mercurials and did not correlate with the clustering of compounds. This precondition was ideal to examine the specific transcriptional changes that were common to all HDACi and that distinguished mercurials from the former group.

It was particularly striking that some mercurials had little to no effect on gene expression deregulation, even though the concentrations used reached the limit of cytotoxicity. This may be due to the fact that mercurials can react with sulphur centre enzymes pivotal for survival and thus quickly lead to cell death. In order to get an indication whether the cytotoxicity thresholds for these compounds are related to the inhibition of enzymes carrying, e.g., an active cysteine in their catalytic centre, we chose the cysteine protease caspase-3 as a model system. For all six mercurials, we observed enzyme inhibition at concentrations below the BMC selected here, and full inhibition of caspase-3 activity occurred close to the concentration used in our study for the microarray analysis (Fig. 2c). Parallel experiments with HDACi showed that no caspase enzyme inhibition in the concentration ranges relevant for this study (data not shown). Thus, whereas HDACi alter transcription as part of their primary mode of action, they indirectly halt the cell cycle or induce cell death when damage is too severe. In contrast, mercurials inhibit enzymes as their primary mode of action, which may directly kill cells without allowing new transcription to occur, if a certain threshold is exceeded. Alternatively, this may lead to cell stress, associated with altered transcription.

### Identification of HDACi consensus genes

As we had obtained information on the transcriptional changes triggered by the six compounds, all linked by their capacity to inhibit HDACs, we questioned whether a common (signature) effect of HDACi on neuro-differentiation of human stem cells can be identified. We determined for each DEG the number of HDACi and of mercurials that regulated it. For instance, 64 genes were up-regulated by all six HDACi; 17 were down-regulated. Amongst these, 18 were regulated by all HDACi, but no mercurial; 24 genes were regulated by at least 5 HDACi and at least 4 mercurials (of those 20 up and down). The latter group may be used as general developmental toxicity indicators. In this way, different consensus regulation groups were identified, and we defined the genes that were regulated by at least 4 HDACi as the HDAC consensus group (independent of being co-affected by mercurials). We identified 405 up-regulated and 190 down-regulated ‘HDACi consensus genes’. Using a similar approach, we identified 53 up-regulated and 12 down-regulated mercurial consensus genes (Suppl. Fig. S3).

### Characterization of the HDACi consensus transcriptome effect in neurally differentiating stem cells

**Fig. 3:**
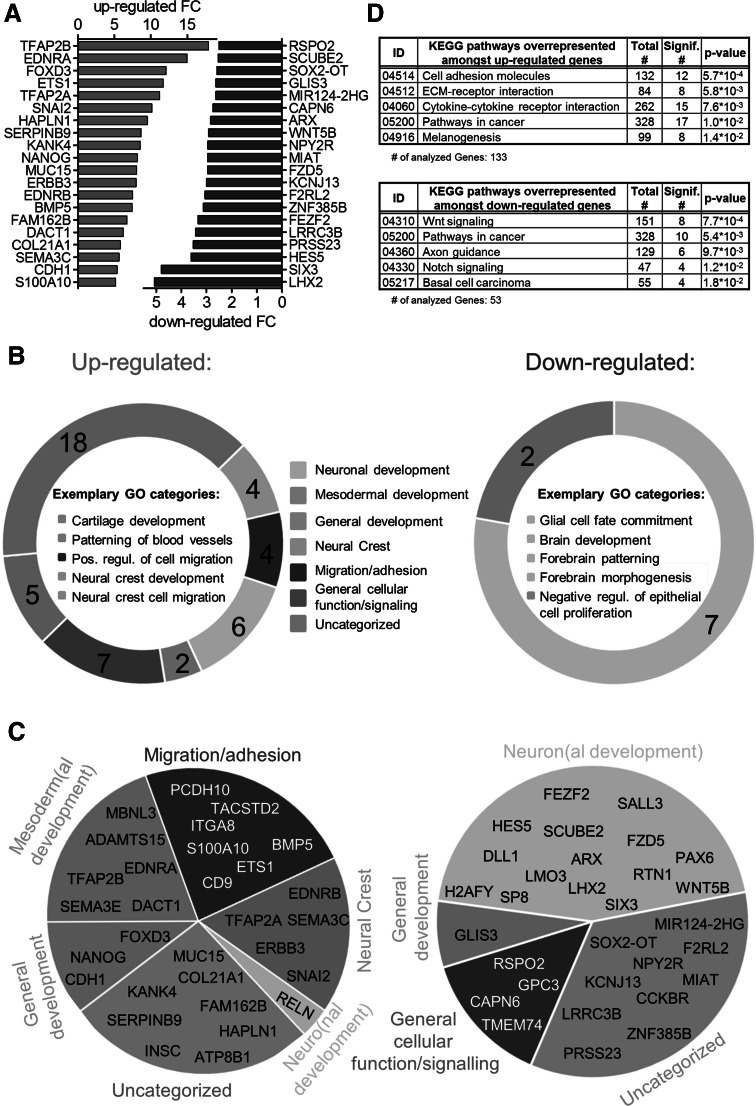
Characterization of the HDACi consensus transcriptome effect in neurally differentiating stem cells. Differentiating cells were treated as indicated in Fig. 1 and used for whole transcriptome analysis. From the differentially expressed genes (DEG), we identified 405 up-regulated and 190 down-regulated ‘consensus genes’, each of them regulated by at least four HDACi (cut-off FC > 1.5). For each consensus gene, the mean fold change (FC) of all 6 HDACi was calculated and used for further analysis (detailed data are shown in supplemental material). **a** The top 20 up- and down-regulated consensus genes are displayed. **b** The gene ontology (GO) categories overrepresented amongst up- and down-regulated consensus genes (*p* < 0.05) were classified into 7 superordinate cell biological processes: neuro(nal development), mesoderm(al development), general development, migration/adhesion, neural crest, general cellular function/signalling and uncategorized and presented as ring diagram to visualize the relative distribution. The number of GO categories in each group is indicated. **c** The top 30 up- and down-regulated consensus genes were classified into 7 superordinate cell biological processes. **d** KEGG pathways overrepresented amongst consensus genes were identified and the five with the lowest *p* values (all with *p* < 0.02) are displayed. The numbers of total genes and the numbers of HDACi consensus genes are shown for each pathway

The top 20 transcripts up-regulated by HDACi comprised neural crest transcription factors (TFAP2A, FOXD3; >10-fold regulation) and transcripts that are expressed in the mesodermal lineage (TFAP2B, ENDRA, DACT1). We also observed a relative up-regulation of the pluripotency gene Nanog (fivefold) compared to cells treated only with solvent. As Nanog belongs to the genes strongly down-regulated in the UKN-1 system under control conditions (Balmer et al. 2012), this latter finding may indicate that a subpopulation of cells remained closer to the pluripotent state, i.e. did not form neuroepithelial cells. Amongst the top 20 down-regulated HDACi consensus transcripts, a broad array of neuronal transcription factors was found (with relative fold changes of 2–3) (Fig. 3a).

To obtain an overview of the main biological processes affected by HDACi, the gene ontology terms (GOs) overrepresented amongst up- and down-regulated consensus genes (*p* < 0.05) were grouped into 7 superordinate cell biological processes (e.g. neuro(nal development), mesoderm(al development)) and the number of GOs in each category was counted. More than 50 % of the up-regulated GO categories were associated with mesoderm/mesodermal development (18 categories, e.g. cartilage development), migration/adhesion (4 categories, e.g. positive regulation of cell migration) and neural crest (4 categories, e.g. neural crest development, neural crest cell migration). This was indicative of erroneous development away from central nervous system/forebrain formation that is usually observed in UKN1 (Balmer et al. 2012, 2014). This was consistent with the observation that the GO categories overrepresented amongst the down-regulated DEG were mainly associated with neuronal development (7 out of 9 categories, e.g. brain development, forebrain patterning and glia cell fate commitment) (Fig. 3b).

This pattern was confirmed by the top 30 most highly regulated individual genes: the group of up-regulated genes contained neural crest markers and transcripts that are expressed in the mesodermal lineage. Amongst the top 30 down-regulated HDACi consensus transcripts, we identified crucial markers for neurodevelopment such as LHX2, HES5, SIX3 (homeobox protein SIX3; crucial for forebrain development; mutations: holoprosencephaly) and ARX (aristaless-related homeobox; mutations are associated with neurological and neurodevelopmental disorders such as lissencephaly, epilepsy, mental retardation) (Fig. 3c).

The KEGG pathways overrepresented amongst the up-regulated genes (cell adhesion molecules, ECM-receptor interaction, melanogenesis) and down-regulated genes (WNT signalling, axon guidance) largely confirmed the above findings on altered cell differentiation/function (Fig. 3d).

Altogether, the biological response of HDACi leads to a loss in efficient neuroectoderm differentiation and induced a shift in differentiation towards neural crest/mesoderm lineage.

### Detection and visualization transcription factor (TF) networks affected by HDACi

To study the changes triggered by HDACi in a systems biology context, we examined whether the changes may be explained by some coordinated action of defined transcription factors or transcription factor networks.

As an initial approach, TF binding sites (TFBS) were identified that were overrepresented in the promoters of the HDACi consensus genes. Amongst the up-regulated genes, binding sites for 109 TF were overrepresented, with SOX9, FOXD3 and LHX3 amongst the most significant (*p* value <10^−16^). Overrepresented TFBS amongst the down-regulated consensus genes suggested regulations by SOX2, TBP, PAX6 and SRY (all *p* values <10^−9^). Notably, many TFBS were overrepresented amongst the up- and down-regulated genes (e.g. SOX9, FOXD3) (Suppl. Fig. S4).

**Fig. 4:**
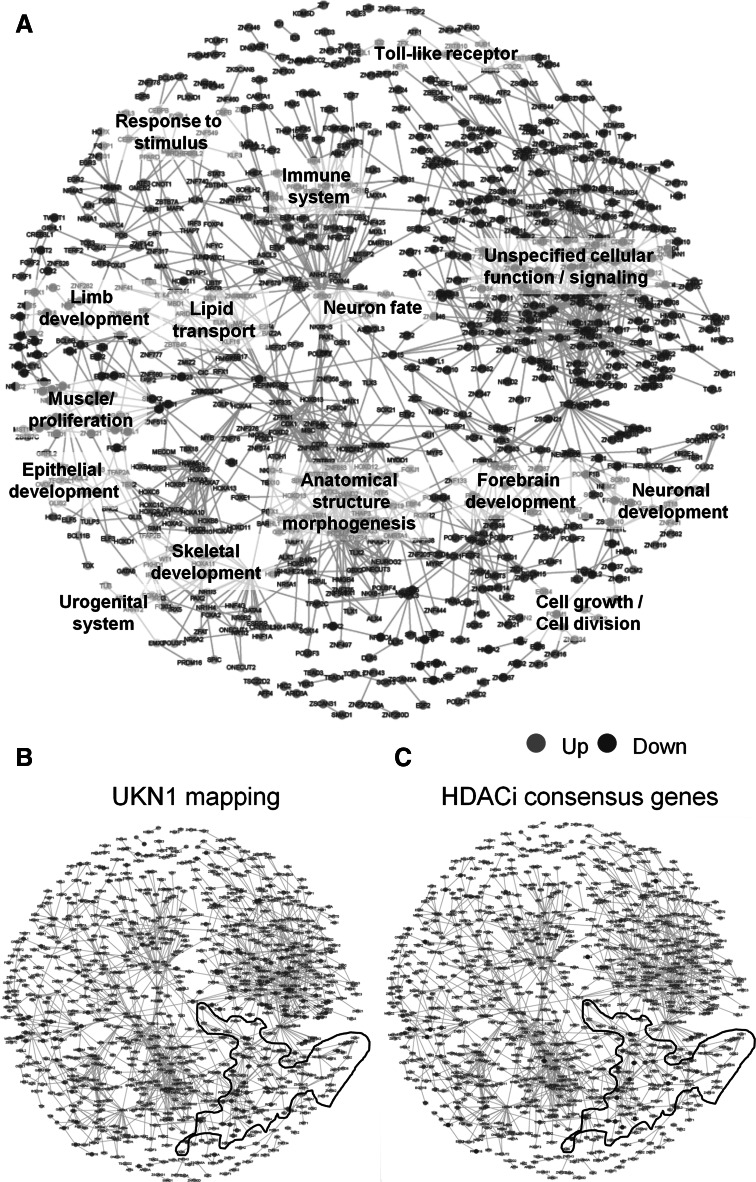
Detection and visualization transcription factor (TF) networks affected by HDACi. **a** The CellNet database (3297 microarray sets from all major tissues) was used to construct a generic human TF network, based on statistical co-expression information and graph-theoretical design principles. Each node represents a TF gene, and each edge suggests co-regulation. The edge length is driven by the number of edges on neighbouring nodes, not by the strength of co-regulation. Nodes are placed according to the Fruchterman–Reingold algorithm. Clusters (coded by same colours) were defined by an optimization algorithm that tries to maximize the modularity of the division of the graph into clusters. Then, GO term overrepresentation analysis was performed for each cluster to identify its biological role, and naming of the 18 clusters is based on these findings. Nodes (*orange*) at the rim of the network displayed in orange have not been assigned to define clusters. **b** The set of genes significantly up-regulated on DoD6 versus DoD0 (*p* < 0.05; FC ≥ 2.0) was retrieved from Balmer et al. (2014), and the TFs of this gene set were marked (*red dots*) in the TF network (see large, scalable version in supplemental material). **c** All TFs were identified amongst the HDACi consensus genes and marked in the TF network (*blue* down-regulated; *red* up-regulated). This diagram indicates, together with information from (**a**), which parts of the TF network are affected by at least 4 of the 6 HDACi used here. The clusters ‘forebrain development’ and ‘neuronal development’ have been encircled for better visualization in **b** and **c** (colour figure online)

In the second approach, we mapped the changes triggered by HDACi to the human TF network. To obtain a platform for this, such a network had to be constructed. Therefore, the CellNet database (Cahan et al. 2014) was used (3297 microarray sets from all major tissues) to build a generic human TF network based on statistical co-expression information. The predicted network exhibited a modular structure that was analysed by defining clusters of TF with increased connectivity. Each cluster was examined for overrepresentation of GO terms in order to identify biological processes predominantly controlled by the genes in the respective cluster. The clusters were assigned names according to the most prominent overrepresented GO terms (Fig. 4a).

This extensive human TF network was used first to visualize main processes relevant to the UKN1 test system. For this purpose, all TFs that were significantly up-regulated during the normal differentiation of human stem cells towards NEP were identified and labelled on the TF map. They were found predominantly in only few clusters. This localization of TF important for UKN1 in sub-networks related to ‘forebrain development’ and ‘neuronal development’ suggested a biological relevance of the mapping. Moreover, localization of many of the remaining TF in ‘general cellular function/signalling’, ‘cell growth/cell division’ and in the mixed cluster named ‘muscle/proliferation’ (constituted of TF coding for these diverse biological processes) reflected the active and dynamic developmental processes occurring in the UKN1 system (Fig. 4b).

After having fully explored the relevance of the TF map and the suggestions it provided of important regulation within the UKN1 system, we used it to map the HDACi consensus genes. These were highly localized to only a few clusters: ‘forebrain development’, ‘neuronal development’, ‘neuronal fate’, ‘muscle/proliferation’ and ‘response to external stimulus’ (Fig. 4c). This indicated that HDACi may share a developmental toxicity mode of action, which involves dysregulation of TF subnetworks pivotal to neuronal development.

Instead of the above direct mapping of the TF found within the HDACi consensus genes, we also used a second bioinformatics approach that may more comprehensively cover the whole set of genes: we tested whether TFs have significantly increased interaction scores for consensus genes, in order to identify the TF that would be responsible for the regulation of the HDACi consensus genes. These TF were then marked on the TF map to study the underlying networks. Again, a relatively selective localization in the clusters of ‘neuronal fate and neurodevelopment’ became evident (Suppl. Fig. S6).

In addition, we calculated tissue specificity of all TF in the TF network (using a two-step procedure of identifying tissue-specific genes, and then identifying the TF amongst them) and produced thus a list of tissue-specific TF (Suppl. Fig. S9). We found that in the UKN1 system, mostly neuronal TF were up-regulated on DoD6. When HDACi consensus genes were examined, neuronal TF were most highly represented amongst down-regulated consensus genes, while ESC TF was most highly represented amongst up-regulated genes (Suppl. Fig. S9B, C).

### Establishment of a classifier for identification of HDACi

**Fig. 5:**
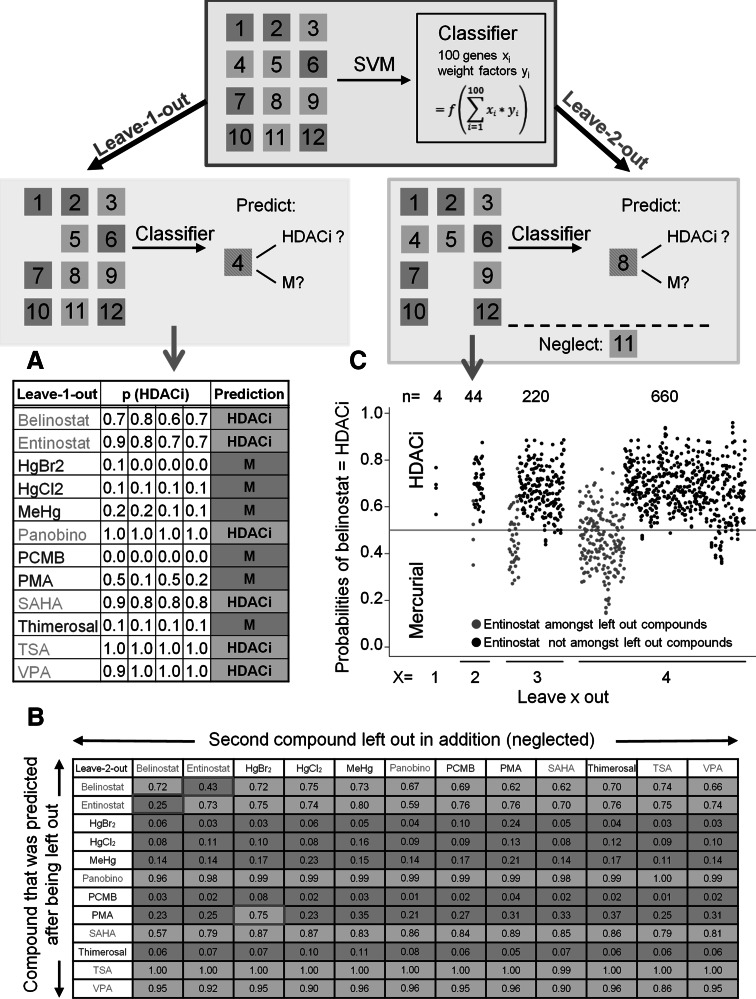
Design of a transcriptome-based classifier to identify HDACi. The scheme on *top* illustrates the setup of a support vector machine (SVM)-based classifier for HDACi. The numbers denote data sets for the 12 toxicants used in this study. *Colours* denote a grouping in mercurials (*blue*) and HDACi (*orange*). Below, the principles of leave-one-out (*left*) and leave-two-out classification are shown. For instance, when chemical-4 is ‘left out’, this means that the other 11 compounds are used to build a classifier according to the rules specified above. Then, it is tested, how well the classification applies to chemical-4. For leave-two-out, the procedure is similar, in that a classifier is built from 10 remaining chemicals to predict one of the compounds left out (e.g. chemical-8). **a** The classifier was validated by a leave-one-out procedure. The calculated probabilities for a toxicant to be an HDACi are shown for each of the four replicate samples, and the overall prediction is shown in the last column. **b** Validation of the SVM classifier by a leave-two-out procedure. The *rows* indicate which compound was left out in addition to the predicted one. The probabilities (prediction) to be an HDACi are presented (for the 144 combinations) as mean of four independent experiments in a cross table. Probabilities of >0.5 predict for a compound to be an HDACi (*red*) and <0.5 predict for a mercurial (*blue*). Incorrect predictions are indicated by a red frame. **c** The SVM-based classifier was used for leave-one-out, leave-two-out, leave-3-out and leave-4-out prediction of belinostat being an HDACi. Predictions were performed for each of the four replicates, and each prediction is represented by a *single dot*. To demonstrate the role of entinostat for the correctness of the prediction, cases in which entinostat was amongst the left-out compounds are marked in *red*. Panobino, Panobinostat (colour figure online)

After the characterization of the set of HDACi consensus genes, we assumed that the relatively stereotypic response triggered by HDACi on the transcript level should form a good basis for the identification of a gene expression-based classifier. A support vector machine (SVM) approach was used to identify weight factors for the 100 probe sets (PS) with the highest variance across all samples (Suppl. Fig. S7). The resulting SVM allowed for discrimination of the six HDACi from the six mercurials (Fig. 5). As initial validation of the classifier, a ‘leave-one-out’ approach was used and 46 out of 48 predictions (12 compounds × 4 replicates) were correct (Fig. 5a).

In a second step, a ‘leave-two-out’ scenario was considered. For this, two of the compounds were left out to be predicted by the ten remaining substances. After averaging the four or five replicates per compound, this resulted in 132 classifications, with 129 being correct. Belinostat was incorrectly classified as a mercurial, when entinostat was left out and vice versa. PMA was classified as HDACi when mercury(II)bromide was left out (Fig. 5b).

The prediction of belinostat as HDACi seemed to represent the most difficult case and was therefore further used to study the effect of entinostat being included in the classifier. In a leave-two-out approach, 44 predictions were made for belinostat being an HDACi (Fig. 5c). While 42 were correct, two were false when entinostat was left out together with belinostat. In a ‘leave-three-out’ simulation, 220 predictions were made, and most of the incorrect ones occurred when entinostat was amongst the left-out compounds. The fraction of false classifications increased in a ‘leave-four-out’ approach, and more than 80 % of these false classifications occurred when entinostat was left out. Therefore, correct classification of belinostat requires that data for entinostat are available for classifier building.

A similar pair of dependent compounds was also found amongst the mercurials. Here the omission of HgBr_2_ leads to the wrong classification of PMA (Fig. 5b). The reason why pairs of compounds depend on each other becomes plausible when their specific position in the PCA is considered (Fig. 1e). Entinostat and belinostat are the two HDACi closest to the three mercurials in the lower left of the PCA plot. It is plausible that omission of either entinostat or belinostat will render classification of the second compound more difficult. Similarly, the mercurial PMA is relatively close to the HDACi VPA (Fig. 1e). This explains why omission of its closest neighbour amongst the mercurials, HgBr_2_, will render the classification of PMA unstable.

### Validation of the transcriptome-based classifier to identify HDACi

After the internal validation by leave-n-out approaches suggested that the full classifier (all 12 compounds) works with good predictivity, we proceeded to independent validation approaches based on external data sets. For this, we used published data obtained from the UKN1 system.

**Fig. 6:**
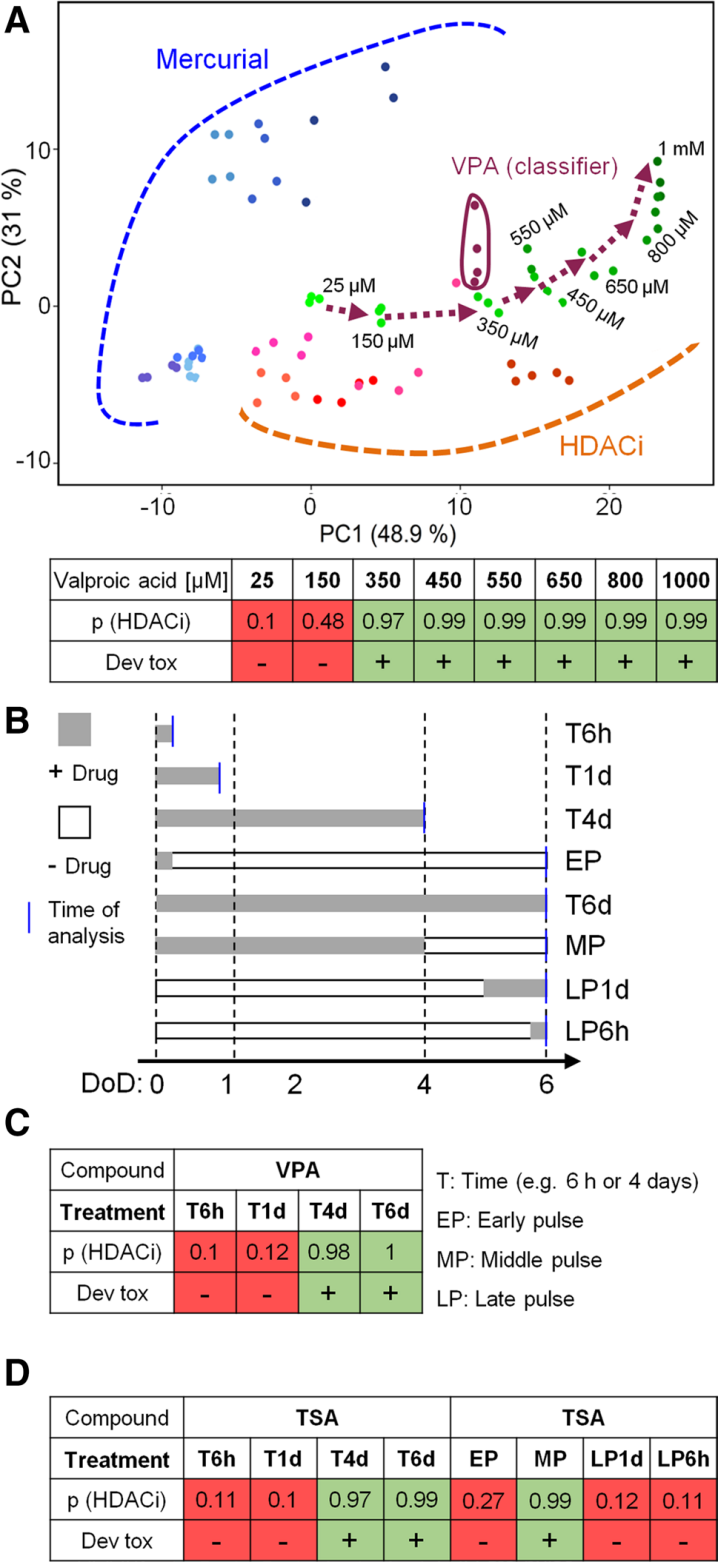
Validation of the transcriptome-based classifier to identify HDACi. **a** Differentiating cells were treated as indicated in Fig. 1 and transcriptome changes of neurally differentiating stem cells induced by HDACi and mercurials are plotted in a PCA (as in Fig. 1e) together with samples treated with 25, 150, 350, 450, 550, 650, 800 and 1 mM valproic acid (VPA) obtained from Waldmann et al. (2014). Each point represents one experiment (=data from one microarray), and the colour coding indicates the compound used in the experiment, mercurials (*blue shades*), HDACi (*red shades*) and VPA legacy data (*green*). The four samples from the present study (VPA classifier) have been encircled for better visualisation. The *purple arrow* indicates the track of transcriptional changes after exposure to increasing concentrations of VPA in the Waldmann et al. (2014) data set. The SVM classifier was applied to this (*green*) data set, and the prediction of VPA, at indicated concentrations (25 µM–1 mM) acting on stem cell differentiation like an HDACi, is shown in the table as a mean of four replicate samples. The *lower row* of the table indicates whether the respective sample triggered developmental toxicity (+) or not (−), according to Waldmann et al. (2014). **b** The diagram shows various schedules of drug exposure. *Grey bars* indicate the period of drug exposure with 600 µM VPA or 10 nM TSA, and *white* *open bars* indicate culture periods in medium without HDACi. The samples were analysed at the times indicated. Exposures of a limited duration relative to the overall experiment were termed ‘pulsed’ treatments, and these were distinguished as early, medium and late pulse according to the exposure scheme. **c**, **d** The tables indicate the calculated probability of VPA or TSA acting like an HDACi when used as described in **b**. Probabilities >0.5 are defined as HDACi classification (*green*), and *p* < 0.5 indicates that the experimental condition did not show a canonical HDAC effect (colour figure online)

First, we used a data set on the effect of different VPA concentrations, for which we had previously identified the range at which developmental toxicity is observed in the UKN1 test (Waldmann et al. 2014). The eight concentrations ranged from 25 to 1000 µM, and the different conditions mapped to largely different positions on the PCA plot used above to show the 12 test compounds of the present study (Fig. 6a). The classifier did not recognize VPA as an HDACi at the two lowest tested concentrations of 25 and 150 µM. At concentrations of 350 µM and higher, classification was excellent, with probabilities close to 100 %. This classification correlated with clinical observations on concentrations that trigger developmental toxicity and with our previous results suggesting that VPA is not affecting neurodevelopment of UKN1 at these low concentrations (Fig. 6a). Thus, the classifier appeared to be specific for concentrations of an HDACi relevant for developmental toxicity and not just any HDACi concentration. Good sensitivity of the classifier was suggested by the fact that VPA concentrations of 350 µM were classified with a probability of 97 % as HDACi, although such a concentration triggered a much smaller transcriptome effect than, e.g. 600 or 800 µM of the compound (Waldmann et al. 2014).

As second approach, we used a data set based on exposure of UKN1 to HDACi for various time periods. We used these legacy data, as previous studies had identified conditions (e.g. short exposure for 24 h) that did not cause developmental effects vs other conditions (e.g. 4- to 6-day exposure) that were associated with toxicity. Short exposures triggered a pronounced transcriptome response, but the regulated genes were different from the ones found after prolonged exposure (Balmer et al. 2012, 2014). We assume that mRNA changes triggered shortly after drug exposure reflected direct actions of HDACi on the cells at the given developmental stage, while longer exposure changed the overall developmental track and thus affected many genes indirectly. Therefore, it is important to consider also the timing of treatment when using a classifier approach as described here (Fig. 6b).

Exposure to VPA for 6 h or 1 day triggered a high number of deregulated genes (Balmer et al. 2014), but this pattern of transcriptome change did not allow classification as HDACi (Fig. 6c). In contrast, exposure for 4 and 6 days resulted in an excellent classification with probabilities close to 100 %. Similar findings were obtained for TSA, a second HDACi. In this case, the time window of exposure was varied in even more conditions, and in all cases that led to developmental toxicity, the classifier had a high value. Conditions that led to other types of transcriptome changes (reversible and not associated with developmental toxicity) resulted in a low probability value from the classifier (Fig. 6d).

In conclusion, correct classification required both a relevant concentration and a relevant exposure period and time window. These two requirements render the classifier relatively specific for developmental toxicity of HDACi, as opposed to other biological effects common to this group of drugs.

### Establishment of an optimized classifier based on 8 genes

**Fig. 7:**
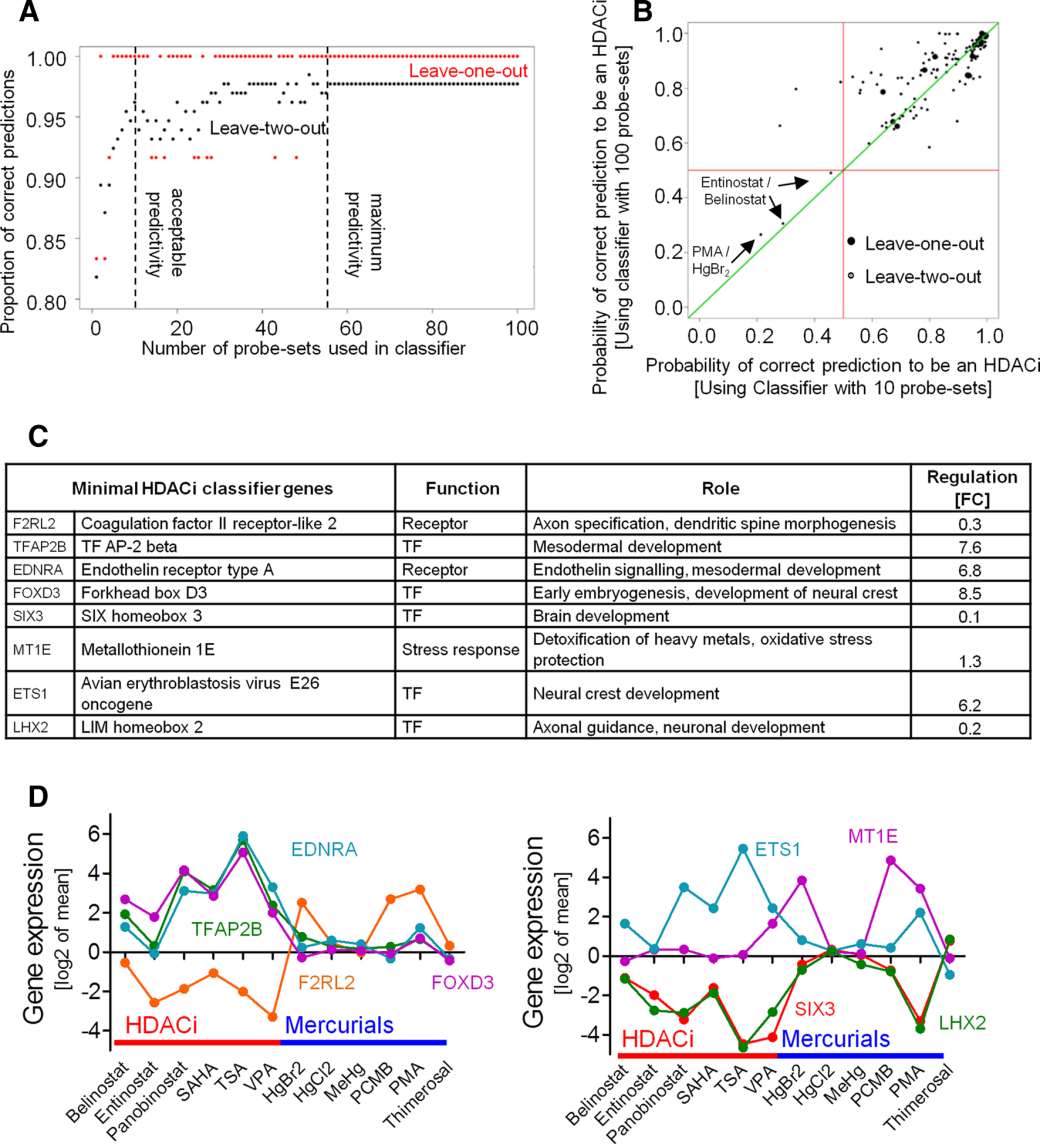
Establishment of an optimized classifier based on 8 genes. **a** Initially, 100 probe sets (PS) were used for the SVM classifier. For further optimization, the number of PS was continuously reduced by one PS (selected randomly), and for each step, the proportion of correct prediction for a toxicant being an HDACi was calculated using the leave-one-out strategy (*red dots*) and leave-two-out strategy (*black dots*). The thresholds for ‘acceptable predictivity’ and ‘maximum predictivity’ are indicated by a *dashed line*. **b** The results of probability predictions for a toxicant being an HDACi determined by a 100-PS-based SVM classifier (as described in Fig. 3) were compared with a 10-PS-based SVM classifier (derived from **a**) in a correlation scatter plot. *Note* When an HDACi was predicted to be an HDACi, with *p* = 0.7, the data point was logged at 0.7. When a mercurial was predicted to be an HDACi, with 0.3, the data point was logged at 0.7 (with HDACi prediction as reference point). The results under leave-one-out conditions are presented as large dots and under leave-two-out conditions as *small dots.* For the three leave-two-out wrong predictions, the respective compound pairs are listed. **c** For the genes constituting the minimal HDACi classifier (10-PS, corresponding to 8 genes), function, role and regulation (mean fold change (FC) of all 6 HDACi) are listed (for references see suppl. Fig. S7). **d** The changes in expression of the 8 HDACi classifier genes (from the 10-PS classifier) induced by HDACi (*red*) and mercurials (*blue*) are graphed (colour figure online)

For routine testing, it would be more practical if compound classification could be achieved by analysing a smaller number (<100) of genes than the number used for our classifier. Therefore, the PS used for classification were sorted according to their variance across all compounds (Suppl. Fig. S7), and then, this list of 100-PS was sequentially (starting at the one with lowest variance) reduced in steps of one. Each new classifier was validated by the ‘leave-one-out’ approach to determine how many of the 12 compounds would be correctly predicted. This analysis illustrates that all 12 compounds still remain correctly predicted when the number of PS is reduced to 49. Further reduction to 48 PS and lower led to some false predictions. However, the proportion of false predictions was small (<10 %), and a 10-PS classifier predicted all compounds correctly for the ‘leave-one-out’ concept (Fig. 7a). The apparently discontinuous change of predictivity (e.g. 10-PS classifier better than 48-PS classifier) is due to the procedure chosen here of sequentially leaving out PS from an ordered predefined list. In this list, the weight given to individual PS depended on all other PS in the respective classifier and changed with each step. This explains for instance why leaving out member 48 *plus* 49 produced a better classifier than leaving out only member 49.

Each of the 100 classifiers obtained above was also validated by the leave-two-out approach. The three false predictions displayed in Fig. 5b led to an initial predictivity of 97.7 %. This remained constant up to a reduction to a 58-PS classifier (threshold of maximum predictivity) and gradually got worse. For the 10-PS classifier, 96.2 % predictivity was obtained, and afterwards the quality deteriorated rapidly. Thus, 10-PS were considered the limit for building a classifier of acceptably high and robust predictivity (Fig. 7a).

The quality of the 100- and the 10-PS-based classifiers was compared in detail for all leave-one-out and leave-two-out predictions (Fig. 7b). While both classifiers worked well for the leave-one-out approach, the 100-PS classifier was superior for some of the leave-two-out predictions. For example, the three compounds in the upper left square of Fig. 7b were only correctly predicted by the 100-PS classifier, while three false predictions were the same for both classifiers. Overall the correlation was high enough for further use of the small classifier in other studies and additional validation experiments.

The 10-PS of the optimized classifier correspond to eight genes (Fig. 7c), five of which are transcription factors. Three of the genes are down-regulated by HDACi, and four are strongly up-regulated. In general, the behaviour of each of these genes differed between HDACi and mercurials, although some heterogeneity was evident also within each group of toxicants (Fig. 7d). Relatively little intra-group variation was observed for FoxD3. This gene would ‘on first sight’’ have been sufficient alone to classify HDACi and mercurials (all of the HDACi showed higher expression levels than the mercurials). However, such a simplified approach would neglect the problem of multiplicity of testing and statistical variation associated with each measurement. This means that when testing 50,000 PS as potential classifiers for 12 compounds, there is a chance to obtain a profile such as the one for FoxD3. In contrast, an eight-gene classifier will be more robust and provides a statistically sound basis for classification under real-life conditions and with additional compounds being measured.

## Discussion

The present study was performed with the stem cell-based test system UKN1 that recapitulates neural induction during a six-day process. For the classification study, we used six histone deacetylase inhibitors (HDACi), because they are acting by similar molecular mechanism (Khan et al. 2008). The goal of the classification study was to test whether the HDACi can be identified and whether they can be differentiated from six compounds containing mercury (‘mercurials’) and known to act by heterogeneous mechanisms. The lack of overlap between the transcriptome changes of mercurials (only 2 PS altered by > 4 compounds) gives evidence that the six analysed mercurials do not represent a homogeneous group of compounds all acting by similar mechanisms; this corresponds to the information from the PCA plots, where the mercurials cluster at two distinct positions in the principle component space (Fig. 1b). The main finding of the study was that a support vector machine-based algorithm correctly predicted each of the 12 compounds. The robustness of the classification is further suggested by the high fraction (48 out of 50) of correctly predicted individual replicates (each represented by one microarray), in addition to the fact that the prediction of each compound—defined by the mean value of the four to five biological replicates per compound—was correct.

A second outcome of our study was the identification and characterization of HDACi consensus genes for UKN1 standard test conditions. All six HDACi deregulated altogether 81 genes, and nearly six hundred were deregulated by at least four HDACi (405 up; 190 down). However, in this context, it is important to revisit the concept of such consensus genes. In earlier studies, an overlap in the response to HDACi has been interpreted as evidence for the existence of an HDACi response pattern (Jergil et al. 2009; Kultima et al. 2010). Such ‘HDAC genes’ were also confirmed upon short exposure to VPA in an embryonic stem cell test system (Theunissen et al. 2012). However, it became clear that HDACi can change up to 10–20 % of the genes of the genome, and it was shown earlier that closely related hESC-based systems responded very differently to VPA (Krug et al. 2013). Moreover, the response triggered by VPA in the UKN1 system showed hardly any overlap with HDACi responses in mESC or tumour systems (Copp et al. 2003; Harris and Juriloff 2007). Thus, the concept of ‘consensus genes’ needs to be refined: (1) they are specific to the cell type studied; (2) they are specific (with a given cell type and drug) to the exposure time at which they are measured (Balmer et al. 2014); (3) they change with drug concentrations, at least when the drug reaches a cytotoxic level (Waldmann et al. 2014); and (4) they mainly reflect the changed differentiation state of the cells after long-term exposure. The high number of HDACi consensus genes identified here gives thus a good description of the altered cellular state, which deviated from the normal endpoint of UKN1 and which is diverted in similar ways by all HDACi.

The present data go beyond our previous findings that TSA and VPA (HDACi with a >10,000-fold difference in potency and with little structural similarity) shared a large proportion (about 70 %) of their regulated probe sets. Analysis of the overlap of the genes up-regulated by the two HDACi TSA and VPA had suggested that HDACi trigger the differentiation of UKN1 cells to several other (unwanted) cellular lineages, such as the cardiovascular system, neural crest, skeletal system and glands, instead of neuroectoderm. Such assumptions were corroborated here. Our new data provide a more solid basis for HDACi consensus genes in the UKN1 system than older data obtained with VPA. Of the six marker genes used previously to further characterize the effects of VPA, only two (Pax6 and Nanog) are in the new set of consensus genes. For instance, OTX2, used in our previous studies on VPA and TSA (Balmer et al. 2014), was here indeed down-regulated by VPA, TSA and PMA, but not by the others HDACi.

With respect to developmental defects modelled by the UKN1 system, we examined whether any of the HDACi consensus genes are known for disturbances of neural tube formation from mouse knockout studies and other experimental evidence. We found that two (GLI3 and NF1) of the down-regulated consensus genes and several of the up-regulated ones are indeed associated with neural tube formation pathology (Copp et al. 2003; Harris and Juriloff 2007, 2010).

Concerning the specificity of the consensus genes, 151 (121 up-regulated + 30 down-regulated) of them were not regulated by any mercurial, and 185 additional ones were only affected by one of the mercurials. This indicates a distinct difference between developmental dysregulation triggered by the group of HDACi and other chemicals. On the other hand, there were also few genes that were regulated by all HDACi and at least four mercurials. This small group of genes may be robust indicators of a generally disturbed development in the UKN1 system and could be considered as compound-independent biomarker candidates. The up-regulated ones comprised: BAG2, COL1A2, GABRB3 (link to several neurodevelopmental diseases), GREM1 (control of organogenesis, BMP antagonist), PHLDA2 (placenta growth, imprinted, tumour suppressor), TFAP2A (neural crest development) and NQO1 (oxidative stress). The two down-regulated ones were NCALD (neurocalcin delta, neuronal calcium sensor) and PRSS23 (ovarian serine protease).

Assignment of identified genes to superordinate biological processes (Falsig et al. 2006), and if possible to key events of adverse outcome pathways (Bal-Price et al. 2015) or other toxicological mechanisms (Grinberg et al. 2014), is an important step from data generation to increased toxicological information. We used here common approaches such as the analysis of gene groups for overrepresented GOs and KEGG pathways. However, we also went one step further by aligning the identified transcript changes with a human transcription factor network constructed specifically for this purpose. Until now, KEGG pathways cover TF networks only to a limited extent, and further refinement of this approach (Rahnenfuhrer and Leist 2015) may lead to the identification of subnetworks as regulatory principles explaining waves of transcript changes and windows of sensitivity to toxicants during defined developmental phases (Kuegler et al. 2010; Zimmer et al. 2011).

An important question is at which concentration the cells of the UKN1 test system should be exposed to test compounds to guarantee correct classification. Previous studies have shown that relatively small increases in concentrations may have massive consequences on the numbers of deregulated genes (Krug et al. 2013). Moreover, additional cell death-associated genes become deregulated when cells are exposed to cytotoxic concentrations (Waldmann et al. 2014). Theoretically, cell death-associated genes may compromise classification when exposure is performed with cytotoxic concentrations. On the other hand, critical genes may not yet be de-regulated at too low concentrations which might lead to false negatives. To avoid such problems, it is current practice to perform a concentration range finding study with the aim to identify the highest non-cytotoxic concentration [benchmark concentration, (Krug et al. 2013)], i.e. the concentration that marks the transition between the non-toxic range and the cytotoxic concentration range of a chemical. However, this procedure is technically challenging, because the cytotoxic range may vary from experiment to experiment. Variability of the benchmark concentration of 25 % or even more is possible. For steep concentration response curves, this may lead to substantial differences in the number of deregulated genes. Due to experimental variability, it may, for instance, occur that a concentration identified as benchmark in the concentration finding test causes a higher or lower cytotoxicity in the main study for the gene expression analysis. So far it has been unclear whether testing at higher concentrations than the benchmark concentration leads to data that are useless for hazard identification. To systematically address this question, we used a set of concentration-dependent gene array data where VPA has been tested at eight concentrations between 25 and 1000 µM. In previous comprehensive studies, it has been shown that 25–125 µM VPA corresponds to a concentration range of ‘tolerance’ where only weak gene expression responses occur and the development of the cells is not compromised (Waldmann et al. 2014). Concentrations between 150 and 550 µM represent the ‘teratogenic’ range with clear phenotypical alterations. Concentrations of 800 µM and higher are cytotoxic, and 1000 µM represents the highest concentration where RNA of sufficient quality still could be harvested. As expected, VPA was correctly predicted as an HDACi by the here-established classifier only in the ‘teratogenic’ concentration range. Interestingly, the classifier was negative in the concentration range of ‘tolerance’ where the predicted probability of the classifier decreased from almost 0.97 (350 µM) to 0.10 (25 µM). A surprising result was the correct prediction in the cytotoxic range. VPA at 800 and even 1000 µM leads to a classifier of 0.99 and was therefore equivalent compared to the ‘teratogenic’ range. Obviously, a ‘dilution effect’ by cytotoxicity associated genes did not occur to a relevant extent. This result may be of high practical relevance, because it supports the recommendation to perform the exposure for gene array experiments at relatively high concentrations; exceeding the benchmark concentration may not be as critical as hitherto expected. On the other hand, too low concentrations, where less than 300 genes are deregulated, should be interpreted with caution. It should, however, be considered that a systematic concentration-resolved classification study has so far only been performed for a single compound (VPA) and further analyses are required before general conclusions can be drawn.

The UKN1 test system includes a 6-day period for exposure to test compounds. For routine testing, shorter exposure periods would be convenient. Therefore, we performed compound washout studies where the UKN1 cell system was exposed only during the first 6 or 24 h (early pulse) to HDACi, followed either by direct analysis or by an incubation period without test compound up to day 6. In both cases, the classifier did not recognize the compounds as HDACi. Also exposures during the last 24 or 6 h of the 6-day differentiation period (late pulse) were insufficient. Only 4-day exposure followed by 2 days washout (medium pulse) resulted in sufficient irreversible expression alterations to correctly classify the test compounds. This may be the period associated with the largest epigenetic changes (Balmer and Leist 2014; Weng et al. 2012, 2014). In conclusion, critical developmental time windows occur during DoD1—4. This period must be covered by compound exposure to guarantee a sufficient sensitivity.

Stepwise reduction in the number of PS demonstrates that a classifier based on only 10-PS still correctly predicts all 12 compounds if the ‘leave-one-out concept’ is used. These 10-PS correspond to only eight genes, because the classifier selected two PS for each of the genes transcription factor AP-2 beta (TFAP2B) and endothelin receptor type A (EDNRA). At a first glance, it may be surprising that only one gene (FOXD3) is in the overlap of the eight-gene classifier (Suppl. Fig. S7) and the HDACi consensus signature (Fig. 3, suppl. Table S2). However, this is a well-known phenomenon of high-dimensional data (Fan 2006). Several classifiers can be constructed that are based on completely different sets of genes but nevertheless perform equally well. It should also be considered that the eight-gene classifier was obtained by a multivariate algorithm and preselection of probe sets based on individual variances, whereas the consensus list is based on simple overlap analysis of all genes up- or down-regulated by all HDACi. Differentiation between HDACi and mercurials is relatively easy in the present set of data and could even be achieved on the basis of only single genes, such as FOXD3 (Fig. 7). However, the eight-gene-based algorithm guarantees more stability when additional compounds are tested.

In conclusion, an eight-gene-based classifier allows the identification of HDACi in a human stem cell-based in vitro system. In future, it will be of interest to study whether further signatures can be identified that can be linked to specific mechanisms of developmental neurotoxicity, and whether such systems can replace animal experiments (Hartung and Leist 2008), by offering higher predictability, as envisioned by the landmark document of the national research council of the USA (Leist et al. 2008b).

## Electronic supplementary material

Supplementary material 1 (PDF 1261 kb)

Supplementary material 2 (XLSX 38127 kb)

Supplementary material 3 (XLSX 342 kb)
