# Supplementary material for: A transcriptome-based classifier to identify developmental toxicants by stem cell testing: design, validation and optimization for histone deacetylase inhibitors
Source: Arch Toxicol. 2015 Aug 14;89(9):1599–618. doi: 10.1007/s00204-015-1573-y (PMC4551554; doi:10.1007/s00204-015-1573-y)
Supplement: Supplementary file 1 — Supplementary material 1 (PDF 1261 kb) [file 204_2015_1573_MOESM1_ESM.pdf]

Supplementary Material

A transcriptome-based classifier to identify developmental toxicants by stem cell testing: design, validation, and optimization for histone deacetylase inhibitors

Eugen Rempel[1, 2], Lisa Hoelting[3,4*], Tanja Waldmann[3], Nina V. Balmer[3], Stefan Schildknecht[3], Marianna Grinberg[1], John Antony Das Gaspar[5], Vaibhav Shinde[5], Regina Stoebe[6], Rosemarie Marchan[6], Christoph van Thriel[6]Thriel[5], Julia Liebing[6], Johannes Meisig[7, 8], Nils Blüthgen[7,8], Agapios Sachinidis[5], Jörg Rahnenführer[1#], Jan G. Hengstler[6#], Marcel Leist[3#]*

Archives of Toxicology

[1]TU Dortmund University, Department of Statistics, 44139 Dortmund, Germany
[2]Centre for Organismal Studies, Heidelberg University, 69120 Heidelberg, Germany
[3]University of Konstanz, Department of in vitro toxicology and biomedicine, 78457 Konstanz, Germany
[4]Konstanz Graduate School Chemical Biology KORS-CB, University of Konstanz, 78457 Konstanz, Germany,
[5]Institute of Neurophysiology and Center for Molecular Medicine Cologne (CMMC), University of Cologne, 50931 Cologne, Germany
[6]Leibniz Research Centre for Working Environment and Human Factors at the Technical University of Dortmund (IfADo), 44139 Dortmund, Germany
[7]Institute of Pathology, Charité-Universitätsmedizin, 10117 Berlin, Germany
[8]Integrative Research Institute for the Life Sciences and Institute for Theoretical Biology, Humboldt Universität, 10115 Berlin, Germany
***shared first authorship, #shared senior authorship**

Table of Contents

Supplementary Figure S1: Literature data on toxicological effects of mercurials relevant for developmental toxicity (DT) or developmental neurotoxicity (DNT). page: 2
Supplementary Figure S2: Determination of the benchmark concentration (BMC) used for toxicant testing on microarrays. page 4
Supplementary Figure S3: Identification of HDACi consensus genes. page: 5
Supplementary Figure S4: Transcription factor binding sites (TFBS) overrepresented amongst up- and down-regulated HDACi consensus genes. page: 6
Supplementary Figure S5: Visualization of a transcription factor (TF) network. page: 7
Supplementary Figure S6: Identification of transcription factors (TF) with HDACi consensus genes enriched among targets. page: 8
Supplementary Figure S7: Transcriptome-based classifier. page: 9
Supplementary Figure S8: Transcriptome-based 100 PS classifier. page: 10
Supplementary Figure S9: Regulation of tissue specific transcription factors (TF). page: 11

Compound	Molecular mechanism	Human evidence for DNT	Reference relevant to DT/DNT
MeHg	Oxidative stress,<br>ROS, lipoperoxidation,<br>GSH inhibition,<br>Disruption of Ca homeostasis,<br>Inhibition of protein synthesis,<br>Apoptosis,<br>Microtubule disruption,<br>Mitochondria toxicity,<br>Mitotic arrest,<br>Reaction with DNA and RNA	+	[1-15]
Thimerosal	Mitochondria toxicity,<br>Reduced oxidative reduction activity,<br>Oxidative stress,<br>Lipid peroxidation,<br>Protein alkylation,<br>Microtubuli disassembly, inhibition of tubuli assembly,<br>Cellular degeneration,<br>Oxidative stress	-	[16-22]
$HgBr_2$ / $HgCl_2$	Cytoskeletal disassembly, tubulin degradation,<br>Disruption of Ca homeostasis,<br>Apoptosis, cell shrinking, chromosome condensation,<br>Mitochondrial dysfunction,<br>Necrosis,<br>Oxidative stress	-	[23-30]
PMA	ATPase inhibition,<br>Peptidases / proteases inhibition	-	[31-33]
PCMBA	Matrix metalloprotease, cleavage and activation	-	[34]

Rempel et al., 2015, Supplementary Fig. S1

Suppl. Fig. S1: Literature data on toxicological effects of mercurials relevant for developmental toxicity (DT) or developmental neurotoxicity (DNT). The six mercurials (methylmercury, thimerosal, mercury(II)chloride, mercury(II)bromide, 4-chloromercuribenzoic acid and phenylmercuric acid), which were used in the present study have one mercury atom in common. A literature research was performed for mechanism relevant for DT and DNT. The heterogeneous mode of action and the potential to cause DNT of the respective mercurials is listed.

List of references:

1.Grandjean, P. and P.J. Landrigan, (2006) Developmental neurotoxicity of industrial chemicals. Lancet. 368. 2167-2178.

2.Grandjean, P. and P.J. Landrigan, (2014) Neurobehavioural effects of developmental toxicity. Lancet Neurol. 13. 330-338.

3.Grandjean, P. and K.T. Herz, (2011) Methylmercury and brain development: imprecision and underestimation of developmental neurotoxicity in humans. Mt Sinai J Med. 78. 107-118.

Continuation list of references:

4.Burbacher, T.M., P.M. Rodier, and B. Weiss, (1990) Methylmercury developmental neurotoxicity: a comparison of effects in humans and animals. Neurotoxicol Teratol. 12. 191-202.

5.Castoldi, A.F., et al., (2008) Human developmental neurotoxicity of methylmercury: impact of variables and risk modifiers. Regul Toxicol Pharmacol. 51. 201-214.

6.Hwang, G.W., (2012) Role of intracellular defense factors against methylmercury toxicity. Biol Pharm Bull. 35. 1881-1884.

7.Atchison, W.D. and M.F. Hare, (1994) Mechanisms of methylmercury-induced neurotoxicity. FASEB J. 8. 622-629.

8.Sarafian, T. and M.A. Verity, (1991) Oxidative mechanisms underlying methyl mercury neurotoxicity. Int J Dev Neurosci. 9. 147-153.

9.Reddy, C.C., R.W. Scholz, and E.J. Massaro, (1981) Cadmium, methylmercury, mercury, and lead inhibition of calf liver glutathione S-transferase exhibiting selenium-independent glutathione peroxidase activity. Toxicol Appl Pharmacol. 61. 460-468.

10.Kuznetsov, D.A., N.V. Zavijalov, A.V. Govorkov, and T.M. Sibileva, (1987) Methyl mercury-induced nonselective blocking of phosphorylation processes as a possible cause of protein synthesis inhibition in vitro and in vivo. Toxicol Lett. 36. 153-160.

11.Castoldi, A.F., et al., (2000) Early acute necrosis, delayed apoptosis and cytoskeletal breakdown in cultured cerebellar granule neurons exposed to methylmercury. J Neurosci Res. 59. 775-787.

12.Sone, N., M.K. Larsstuvold, and Y. Kagawa, (1977) Effect of methyl mercury on phosphorylation, transport, and oxidation in mammalian mitochondria. J Biochem. 82. 859-868.

13.Sager, P.R., R.A. Doherty, and P.M. Rodier, (1982) Effects of methylmercury on developing mouse cerebellar cortex. Exp Neurol. 77. 179-193.

14.Gruenwedel, D.W. and D.S. Lu, (1970) Changes in the sedimentation characteristics of DNA due to methylmercuration. Biochem Biophys Res Commun. 40. 542-548.

15.Gruenwedel, D.W. and M.K. Cruikshank, (1979) Effect of methylmercury (II) on the synthesis of deoxyribonucleic acid, ribonucleic acid and protein in HeLa S3 cells. Biochem Pharmacol. 28. 651-655.

16.Brunner, M., S. Albertini, and F.E. Wurgler, (1991) Effects of 10 known or suspected spindle poisons in the in vitro porcine brain tubulin assembly assay. Mutagenesis. 6. 65-70.

17.Anundi, I., J. Hogberg, and A.H. Stead, (1979) Glutathione depletion in isolated hepatocytes: its relation to lipid peroxidation and cell damage. Acta Pharmacol Toxicol. 45. 45-51.

18.Geier, D.A., P.G. King, and M.R. Geier, (2009) Mitochondrial dysfunction, impaired oxidative-reduction activity, degeneration, and death in human neuronal and fetal cells induced by low-level exposure to thimerosal and other metal compounds. Toxicol Environ Chem. 91. 735-749.

19.Sharpe, M.A., A.D. Livingston, and D.S. Baskin, (2012) Thimerosal-Derived Ethylmercury Is a Mitochondrial Toxin in Human Astrocytes: Possible Role of Fenton Chemistry in the Oxidation and Breakage of mtDNA. J Toxicol. 2012. 373678.

20.Marques, R.C., J.G. Dorea, and J.V. Bernardi, (2010) Thimerosal exposure (from tetanus-diphtheria vaccine) during pregnancy and neurodevelopment of breastfed infants at 6 months. Acta Paediatr. 99. 934-939.

21.Parker, S.K., B. Schwartz, J. Todd, and L.K. Pickering, (2004) Thimerosal-containing vaccines and autistic spectrum disorder: a critical review of published original data. Pediatrics. 114. 793-804.

22.Heron, J., J. Golding, and A.S. Team, (2004) Thimerosal exposure in infants and developmental disorders: a prospective cohort study in the United kingdom does not support a causal association. Pediatrics. 114. 577-583.

23.Xu, F., et al., (2012) Mercury-induced toxicity of rat cortical neurons is mediated through N-Methyl-D-Aspartate receptors. Mol Brain. 5. 30.

24.Szucs, A., C. Angiello, J. Salanki, and D.O. Carpenter, (1997) Effects of inorganic mercury and methylmercury on the ionic currents of cultured rat hippocampal neurons. Cell Mol Neurobiol. 17. 273-288.

25.Araragi, S., et al., (2003) Mercuric chloride induces apoptosis via a mitochondrial-dependent pathway in human leukemia cells. Toxicology. 184. 1-9.

26.Goering, P.L., D. Thomas, J.L. Rojko, and A.D. Lucas, (1999) Mercuric chloride-induced apoptosis is dependent on protein synthesis. Toxicol Lett. 105. 183-195.

27.Shenker, B.J., T.L. Guo, and I.M. Shapiro, (2000) Mercury-induced apoptosis in human lymphoid cells: evidence that the apoptotic pathway is mercurial species dependent. Environ Res. 84. 89-99.

28.Issa, Y., et al., (2003) Mercuric chloride: toxicity and apoptosis in a human oligodendroglial cell line MO3.13. Biomaterials. 24. 981-987.

29.Gutierrez, L.L., et al., (2006) Peripheral markers of oxidative stress in chronic mercuric chloride intoxication. Braz J Med Biol Res. 39. 767-772.

30.Joshi, D., et al., (2014) N-acetyl cysteine and selenium protects mercuric chloride-induced oxidative stress and antioxidant defense system in liver and kidney of rats: a histopathological approach. J Trace Elem Med Biol. 28. 218-226.

31.Pedersen, P.L., (1976) Adenosine triphosphatase from rat liver mitochondria - evidence for a mercurial-sensitive site for the activating anion bicarbonate. Biochem Biophys Res Commun. 71. 1182-1188.

32.Yoshimoto, T., W.H. Simmons, T. Kita, and D. Tsuru, (1981) Post-proline cleaving enzyme from lamb brain. J Biochem. 90. 325-334.

33.Du, P.G., et al., (2001) Rat tripeptidyl peptidase I: molecular cloning, functional expression, tissue localization and enzymatic characterization. Biol Chem. 382. 1715-1725.

34.Warren, M.C., E.A. Bump, D. Medeiros, and S.J. Braunhut, (2000) Oxidative stress-induced apoptosis of endothelial cells. Free Radic Biol Med. 29. 537-547.

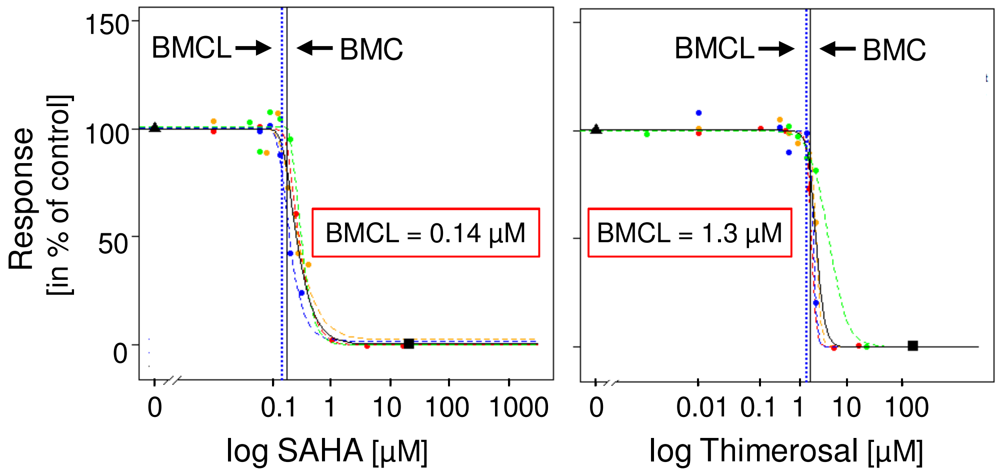

Rempel et al., 2015, Supplementary Fig. S2

Suppl. Fig. S2: Determination of the benchmark concentration (BMC) used for toxicant testing on microarrays. Compounds (here exemplified for the HDACi SAHA and the mercurial thimerosal) were tested at multiple dilutions in the standard UKN1 setup for 6 days. On DoD6, the viability was measured by a resazurin reduction assay, and data were normalized to untreated control samples. Experiments were performed in 3-4 cell preparations (each indicated by a different colour of data point and curves). An average curve (black) was calculated to determine the concentration at which viability was reduced to 90% (=BMC). Then the lower 95% confidence interval of this concentration was calculated (=BMCL). This value was used for all compounds to obtain microarray data (and is referred to as "BMC" in the text).

A

Genes up-regulated by x HDACi

Also regulated by Y mercurials	x = 4	x = 5	x = 6
Y = 0	73	32	16
1	58	41	13
2	42	29	12
3	22	28	16
4	3	12	6
5	0	1	1
6	0	0	0
Sum:	198	143	64

B

Genes down-regulated by x HDACi

Also regulated by Y mercurials	x = 4	x = 5	x = 6
Y = 0	22	6	2
1	53	14	6
2	13	13	3
3	33	14	4
4	3	2	2
5	0	0	0
6	0	0	0
Sum:	124	49	17

C

Genes regulated by mercurials

x =	3	4	5	6
UP	184	51	2	0
DOWN	177	12	0	0

Rempel et al., 2015, Supplementary Fig. S3

Suppl. Fig. S3: Identification of HDACi consensus genes. The UKN1 test system was exposed to 6 HDACi and 6 mercurials (as in Fig. 1) and DEG were identified (see Fig 2B). (A) The genes that were up-regulated by 4, 5, or 6 HDACi were counted (see “sum” line). The gene lists were further differentiated as to whether genes did not affect any mercurial (top line) or they were affected by the indicated numbers of mercurials. (B) The same procedure as in A was applied to down-regulated genes. (C) For a complete overview, the genes up- or down-regulated by at least 3 mercurials were compiled. For the gene list see suppl. Table S2.

A

TFBS overrepresented amongst up-regulated genes	# target gene	p-value
Sox5	300	1.07E-21
SOX9	272	4.12E-20
Foxd3	270	7.85E-20
STAT1	187	7.85E-20
Foxa2	274	7.85E-20
FOXI1	274	9.33E-20
USF1	237	1.32E-19
FOXA1	297	1.83E-19
REL	253	1.51E-18
SRY	313	1.56E-18
CEBPA	286	4.94E-18
HLF	165	5.11E-18
Foxq1	210	6.53E-18
FOXF2	136	9.22E-18
Prrx2	315	1.50E-17
TEAD1	158	1.52E-17
Pdx1	320	2.71E-17
EBF1	261	2.71E-17
Ddit3::Cebpa	164	2.78E-17
MEF2A	190	3.84E-17

B

TFBS overrepresented amongst down-regulated genes	# target gene	p-value
Myf	131	2.29E-20
TBP	132	2.29E-20
MZF1_5-13	149	2.29E-20
SOX9	138	2.29E-20
Arnt	108	2.29E-20
Foxd3	137	4.56E-20
HIF1A::ARNT	134	6.85E-20
Arnt::Ahr	148	3.22E-19
NFYA	96	3.22E-19
RUNX1	148	4.03E-19
Foxq1	112	1.34E-18
FOXO3	147	1.48E-18
FOXI1	136	2.25E-18
Sox17	148	2.30E-18
RELA	105	2.30E-18
Myb	146	2.54E-18
NF-kappaB	114	2.54E-18
Gfi	147	2.85E-18
Nobox	146	3.22E-18
Myc	113	3.38E-18

Rempel et al., 2015, Supplementary Fig. S4

Suppl. Fig. S4: Transcription factor binding sites (TFBS) overrepresented amongst up- and down-regulated HDACi consensus genes. The UKN1 test system was exposed to 6 HDACi (as in Fig. 1) and amongst the DEG, up- and down-regulated HDACi consensus genes were identified (see Fig. S3). The overrepresented TFBS in these sets of genes were determined by using oPOSSUM web tool and JASPAR database.

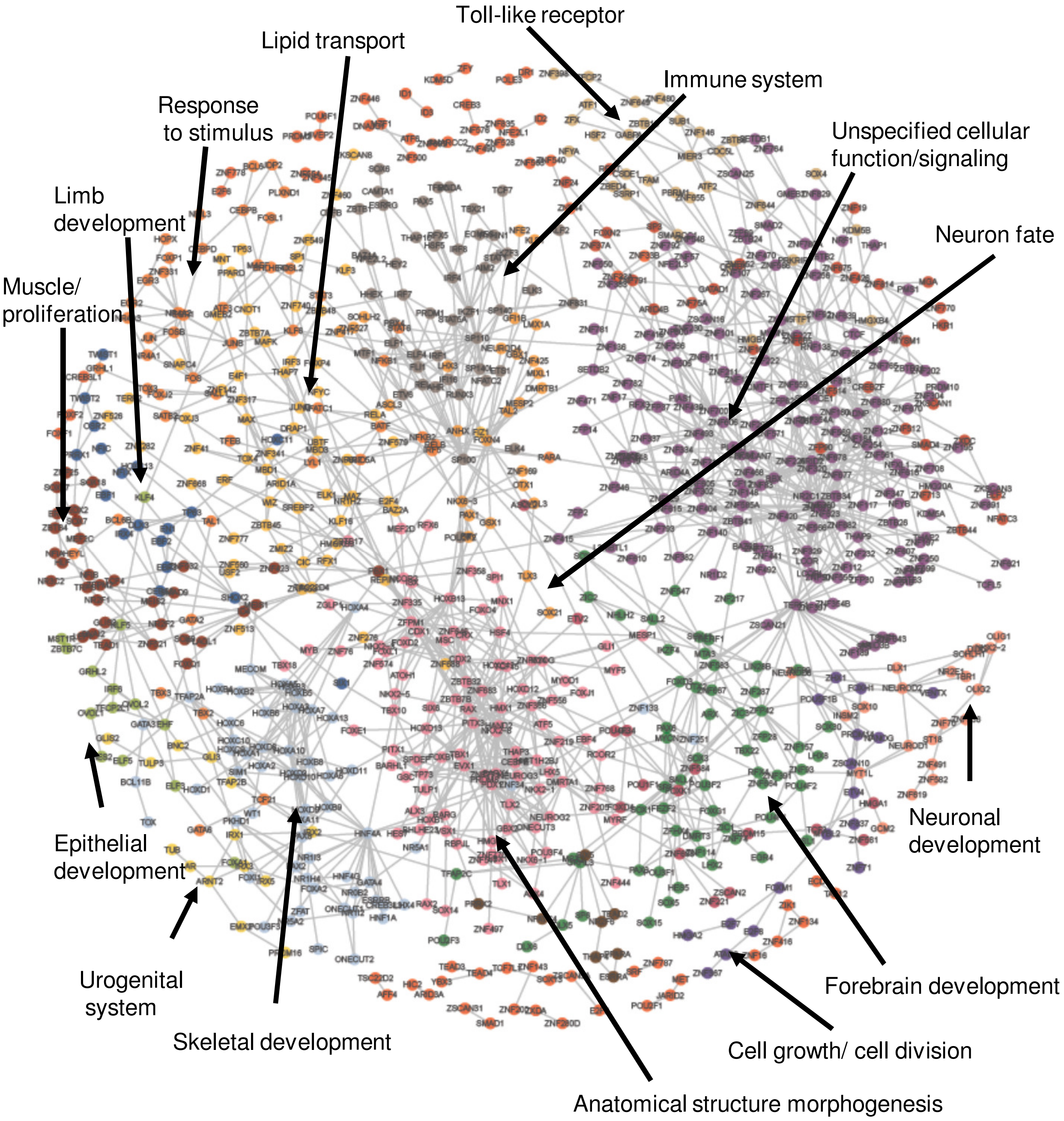

Rempel et al., 2015, Supplementary Fig. S5

Supplementary Fig. S5: Visualization of a transcription factor (TF) network. (A) The CellNet database (2839 microarray sets from all major tissues) was used to construct a generic human TF network, based on statistical co-expression information and graph-theoretical design principles. Each node (n=1000) represents a TF gene, and each edge suggests co-regulation. The edge lengths is driven by the number of edges on neighbouring nodes, not by the strength of co-regulation. Nodes are placed according to the Fruchterman-Reingold algorithm. Clusters (coded by same colours) were defined by an optimization algorithm that tries to maximize the modularity of the division of the graph into clusters. Then GO term overrepresentation analysis was performed for each cluster to identify its biological role, and naming of the 16 clusters is based on these findings.The figure is similar to Fig. 4A, but it allow better reading of all node names.

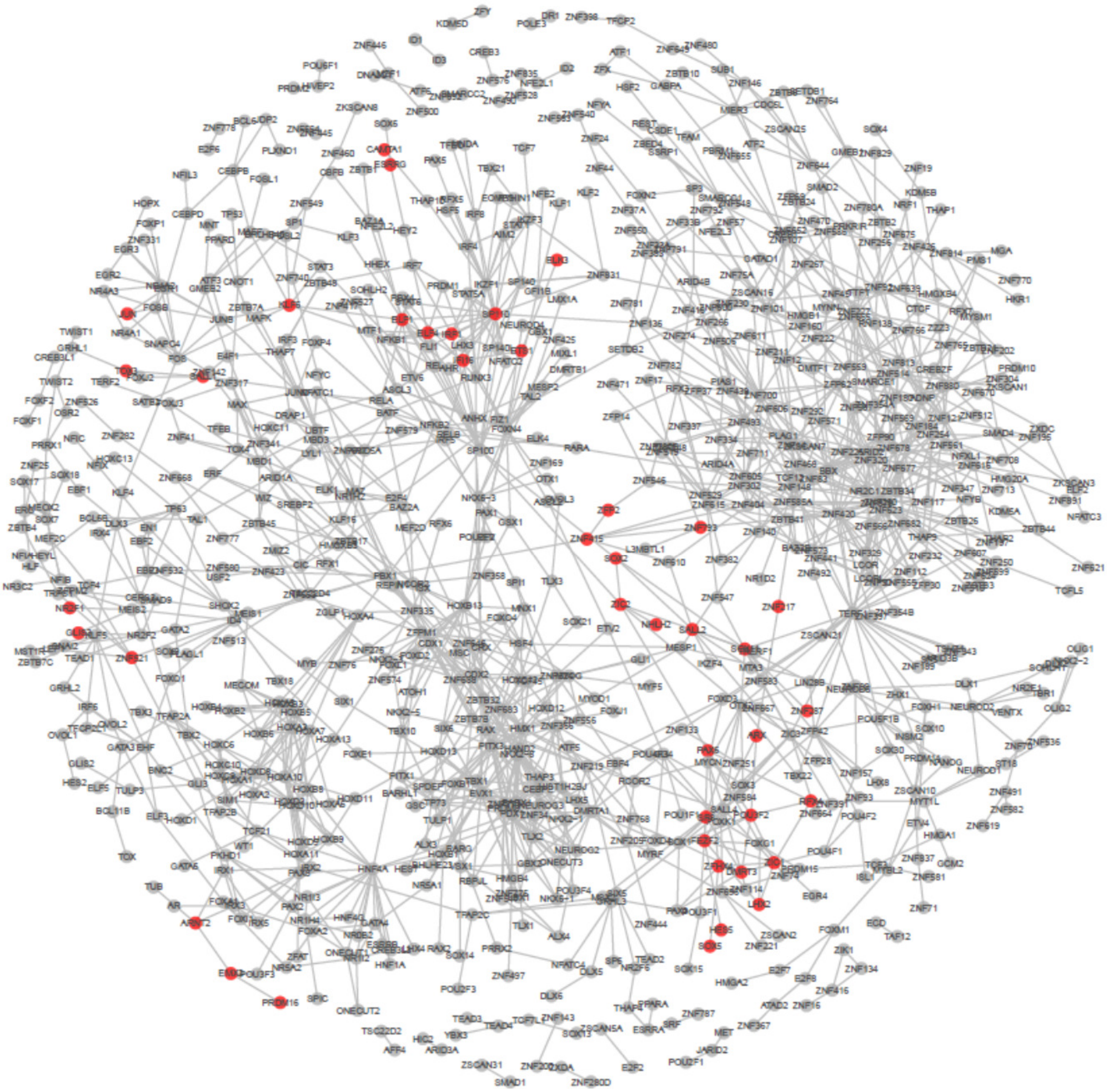

Rempel et al., 2015, Supplementary Fig. S6

Suppl. Fig. S6: Identification of transcription factors (TF) with HDACi consensus genes enriched among targets. An enrichment analysis was performed using the interaction scores generated by the CLR algorithm. For each TF, the scores with the HDACi consensus genes were compared to the distribution of the scores of all genes using Wilcoxon's rank-sum test. TFs with an enrichment p-value less than10-20 were marked red in the network.

Minimal HDACi classififer gene	Regulation [FC]						weight factor γ	Reference relevant to role and function (see also Fig. 7C)
	Belinostat	Entinostat	Panobino	SAHA	TSA	VPA		
F2RL2	0.69	0.17	0.27	0.48	0.25	0.10	0.262	1
TFAP2B	3.79	1.26	17.08	8.90	51.84	5.17	-0.102	2
EDNRA	2.44	0.95	8.69	7.87	60.13	9.90	-0.014	3
FOXD3	6.44	3.46	17.99	7.23	33.41	4.02	-0.26	4
SIX3	0.46	0.25	0.11	0.33	0.05	0.06	0.086	5
MT1E	0.83	1.26	1.26	0.92	1.04	3.12	0.136	6
ETS1	3.13	1.29	11.24	5.37	43.90	5.40	-0.04	7
LHX2	0.45	0.15	0.14	0.27	0.04	0.14	0.094	8

Rempel et al., 2015, Supplementary Fig. S7

Suppl. Fig. S7: Transcriptome-based 10 PS classifier. To calculate classifier value the fold change (FC) of each PS is multiplied with the weighting coefficient γ and summed up over all classifier components (i = 1-8 for 10 PS classifier as shown, i = 1-100 for 100 PS classifier). The offset 0.896 is added.

$$x = \sum_{i=1}^{8} (\gamma_i * FC_i) + 0.896$$

Afterwards, a logistic transformation was applied. $logit(x) = \frac{1}{1+\exp(Ax+B)}$ with A = -2.57 and B = 0.03 (For 100 PS classifier: A = -2.41; B = -0.12). The result is the probability of a compound to represent an HDACi. For the genes constituting the minimal HDACi classifier (10 PS, corresponding to 8 genes), the regulation (of all 6 HDACi) and the weighting factor are listed.

Reference to classifier genes:

1. Zhang, H. and I.G. Macara, (2006) *The polarity protein PAR-3 and TIAM1 cooperate in dendritic spine morphogenesis.* Nat Cell Biol, **8** 227-237.
2. Damberg, M., (2005) *Transcription factor AP-2 and monoaminergic functions in the central nervous system.* J Neural Transm, **112** 1281-1296.
3. Lehmann, L.H., D.A. Stanmore, and J. Backs, (2014) *The role of endothelin-1 in the sympathetic nervous system in the heart.* Life Sci, **118** 165-172.
4. Fairchild, C.L., J.P. Conway, A.T. Schiffmacher, L.A. Taneyhill, and L.S. Gammill, (2014) *FoxD3 regulates cranial neural crest EMT via downregulation of tetraspanin18 independent of its functions during neural crest formation.* Mech Dev, **132** 1-12.
5. Lagutin, O.V., C.C. Zhu, D. Kobayashi, J. Topczewski, K. Shimamura, L. Puelles, H.R. Russell, P.J. McKinnon, L. Solnica-Krezel, and G. Oliver, (2003) *Six3 repression of Wnt signaling in the anterior neuroectoderm is essential for vertebrate forebrain development.* Genes Dev, **17** 368-379.
6. Ito, Y., H. Tanaka, and H. Hara, (2013) *The potential roles of metallothionein as a therapeutic target for cerebral ischemia and retinal diseases.* Curr Pharm Biotechnol, **14** 400-407.
7. Gao, Z., G.H. Kim, A.C. Mackinnon, A.E. Flagg, B. Bassett, J.U. Earley, and E.C. Svensson, (2010) *Ets1 is required for proper migration and differentiation of the cardiac neural crest.* Development, **137** 1543-1551.
8. Marcos-Mondejar, P., S. Peregrin, J.Y. Li, L. Carlsson, S. Tole, and G. Lopez-Bendito, (2012) *The lhx2 transcription factor controls thalamocortical axonal guidance by specific regulation of robo1 and robo2 receptors.* J Neurosci, **32** 4372-4385.

ID	Symbol	Weights γ	ID	Symbol	Weights γ
230147_at	F2RL2	2.12	206588_at	DAZL	5.17
214451_at	TFAP2B	-2.17	236359_at	SCN4B	-1.72
204463_s_at	EDNRA	-1.06	204745_x_at	MT1G	0.692
204464_s_at	EDNRA	-1.61	230895_at	HAPLN1	-1.25
1553394_a_at	TFAP2B	-2.66	230204_at	HAPLN1	-1.36
241612_at	FOXD3	-4.18	205113_at	NEFM	0.768
242054_s_at	SIX3	1.94	228875_at	FAM162B	-5.85
212859_x_at	MT1E	1.54	214265_at	ITGA8	0.078
224833_at	ETS1	-2.64	235666_at	ITGA8	-0.571
206140_at	LHX2	1.33	219179_at	DACT1	-2.37
208581_x_at	MT1X	0.998	201468_s_at	NQO1	2.82
206461_x_at	MT1H	1.07	237056_at	INSC	-1.57
213139_at	SNAI2	-1.39	1555800_at	ZNF385B	0.63
229125_at	KANK4	-1.47	206018_at	FOXG1	-1.52
238878_at	ARX	0.1	209199_s_at	MEF2C	-0.714
221086_s_at	FEZF2	1.22	205430_at	BMP5	-2.44
204326_x_at	MT1X	0.282	215729_s_at	VGLL1	1.13
203789_s_at	SEMA3C	0.0657	228658_at	MIAT	2.89
209160_at	AKR1C3	3.03	1554314_at	C6orf141	0.592
220184_at	NANOG	-10.3	209200_at	MEF2C	-0.755
217165_x_at	MT1F	1.37	208096_s_at	COL21A1	-0.0831
212185_x_at	MT2A	1.69	211456_x_at	MT1HL1	1.53
216336_x_at	NA	0.631	219054_at	NPR3	-0.845
227238_at	MUC15	-1.23	1556378_a_at	LOC440896	0.755
233972_s_at	FEZF2	0.695	210524_x_at	NA	2.06
210302_s_at	MAB21L2	1.18	201012_at	ANXA1	1.48
226213_at	ERBB3	-0.835	237435_at	NA	-1.5
204653_at	TFAP2A	-4.28	243611_at	MICALCL	-7.71
209875_s_at	SPP1	1.28	206029_at	ANKRD1	-0.257
239205_s_at	NA	-7	230493_at	SHISA2	0.722
204273_at	EDNRB	-2.71	230008_at	THSD7A	2.17
203665_at	HMOX1	-1.19	237322_at	MIAT	4.29
206634_at	SIX3	3.03	210729_at	NPY2R	0.902
1555801_s_at	ZNF385B	1.06	204932_at	TNFRSF11B	-2.57
205523_at	HAPLN1	-0.91	229004_at	ADAMTS15	-3.8
243555_at	NA	-1.54	210519_s_at	NQO1	3.77
223044_at	SLC40A1	-1.08	205923_at	RELN	-1.04
206801_at	NPPB	3.02	228367_at	ALPK2	2.93
240055_at	NA	-0.423	219197_s_at	SCUBE2	-0.141
214920_at	THSD7A	0.92	204058_at	ME1	4.33
228347_at	SIX1	-0.744	227241_at	MUC15	1.47
221950_at	EMX2	-1.53	242193_at	MIR124-2HG	0.228
202454_s_at	ERBB3	-2.41	204112_s_at	HNMT	1.75
213629_x_at	MT1F	1.3	209735_at	ABCG2	1.27
209723_at	SERPINB9	-4.99	216235_s_at	EDNRA	-3.25
213894_at	THSD7A	-0.888	210303_at	MAB21L2	4.66
1552521_a_at	TMEM74	0.6	1561101_at	JAKMIP2-AS1	-3.27
1554012_at	RSPO2	1.52	237449_at	SP8	0.75
221245_s_at	FZD5	1.55	203324_s_at	CAV2	2.74
213943_at	TWIST1	-0.0731	205286_at	TFAP2C	-5.4

Rempel et al., 2015, Supplementary Fig. S8

Suppl. Fig. S8: Transcriptome-based 100 PS classifier. To calculate the classifier value, the foldchange of each PS is multiplied with the respective weighting coefficient γ (=weight/100), summed up and further processed as described in suppl. Fig. S7.

A<br>Tissue specific TF		B<br>TF up-regulated at DoD6	C<br>HDACi consensus TF	
		Percentage of tissue-specific TF up-regulated at DoD6 vs DoD0 in untreated cells	Percentage of tissue-specific TF found in HDACi consensus genes	
Tissue	Number (N)		UP	DOWN
Neuron	48	17% (1)	3% (4)	12% (1)
ESC	96	13% (2)	11% (1)	9% (2)
Colon	31	7% (4)	4% (2)	4% (3)
Ovary	85	12% (3)	4% (2)	4% (3)
MuscleSkel	104	4% (5)	3% (4)	2% (5)

Numbers in brackets indicate rank position in list

Rempel et al., 2015, Supplementary Fig. S9

Suppl. Fig. S9: Regulation of tissue specific transcription factors (TF). (A) Tissue-specific TF were determined as follows. First, the CellNet tissue data was used to calculate the average expression of each TF in each tissue. Subsequently, the average expression was binned into 10 quantiles from 0 (lowest) to 9 (highest). To determine TF with preferential expression in one tissue, we computed the tissue specificity index t proposed in (Yanai et al. 2005).

$$t_j = \frac{\sum_{i=1}^{N} 1 - x_{ij}/max_i\,(x_{ij})}{N-1}$$

In the above formula, i runs over all tissues, N is the number of tissues, x_{ij} is the expression quantile of TF j in tissue i. The top 50% TF with the highest specificity index (t) were then assigned to the tissue in which they were expressed most highly. This resulted in 671 TF with 'tissue specific expression'. The absolute number for five tissues are listed.

(B) The percentage of tissue specific TF, which were up-regulated at DoD6 vs DoD0 in untreated cells was determined. (C) The percentage of tissue-specific TF found in up- and down-regulated HDACi consensus genes was counted.
